# A monofunctional-like mutant of DNA glycosylase NTHL1 changes the dynamics of DNA repair during acute oxidative stress

**DOI:** 10.1016/j.jbc.2026.111332

**Published:** 2026-02-27

**Authors:** James Haslam, Natalie Rudolfova, Kaixin Zhou, Evert Homan, Ann-Sofie Jemth, Maurice Michel, Thomas Helleday, Oliver Mortusewicz

**Affiliations:** 1 Science for Life Laboratory, Department of Oncology-Pathology, Karolinska Institutet, Stockholm, Sweden; 2 Center for Molecular Medicine, Karolinska Institutet and Karolinska Hospital, Stockholm, Sweden

**Keywords:** NTHL1, DNA repair, base excision repair, DNA glycosylase, oxidative stress

## Abstract

Bifunctional DNA glycosylases, which initiate the base excision repair (BER) of oxidized bases, act by first excising the base and then incising the DNA backbone. *In vitro*, these enzymes are often rate-limited by their apurinic/apyrimidinic (AP)-lyase activity; however, the significance of this step in cells has remained unclear, because AP-endonuclease 1 (APE1) can efficiently bypass this step. To analyze the importance of the AP-lyase activity of NTHL1, we rationally designed and characterized a monofunctional-like NTHL1 mutant with glycosylase activity but profoundly impaired AP-lyase activity using complementary biochemical and microscopy-based assays. Mechanistically, we demonstrated that the monofunctional-like NTHL1 mutant generates abasic sites (AP sites) but, despite lacking effective AP-lyase activity, remains AP site bound. This creates competition with APE1 for engagement of AP sites. Moreover, cells expressing the monofunctional-like NTHL1 mutant accumulated more AP sites, retained higher levels of XRCC1 foci and displayed heightened sensitivity to acute oxidative stress. Live-cell assays further revealed increased NTHL1 accumulation at laser-induced DNA damage sites and increased chromatin bound immobility during oxidative stress, with mobility restored after a repair period. In contrast, a catalytically inactive NTHL1 mutant was recruited less strongly but remained chromatin-bound for a longer time. Thus, in contrast to the monofunctional-like NTHL1 mutant, the bifunctional NTHL1 limits BER intermediate retention and enables timely hand-off to downstream enzyme APE1. Ultimately, disrupting the AP-lyase ability of NTHL1 disrupts BER pathway flux and affects chromatin engagement during oxidative stress.

The conserved base excision repair (BER) pathway is responsible for the repair of damaged DNA bases, particularly those arising from oxidation due to reactive oxygen species (1, 2). Cancer cells often exhibit elevated reactive oxygen species levels, driven in part by oncogene-induced metabolic changes that can promote oxidative DNA damage (3, 4, 5, 6). Characterizing the importance and role of BER proteins to genome stability in normal and cancer cells has been a longstanding focus of research. DNA glycosylases, which can be described as monofunctional or bifunctional, are responsible for the initiation of the multistep BER process by recognizing and excising the damaged nucleobase (7). Although many of the human DNA glycosylases are monofunctional, catalyzing only the hydrolysis of the glycosidic bond which removes the damaged nucleobase and leaving an apurinic/apyrimidinic site (AP site), some have been described to be bifunctional enzymes (8, 9).

The human endonuclease III-like protein I (NTHL1) is an example of a bifunctional DNA glycosylase, which recognizes and removes a range of oxidized pyrimidine derivatives (10, 11, 12, 13). Like other bifunctional DNA glycosylases, it can also cleave the phosphodiester bond of the DNA backbone with its apurinic/apyrimidinic-lyase (AP-lyase) activity within the same active site. While the NEIL1/2 DNA glycosylases perform β,δ-elimination to generate the intermediate single-strand break (SSB), channeling BER through a PNKP1-dependent pathway (14), NTHL1 can only perform β-elimination. This generates a 3′-phospho-α,β-unsaturated aldehyde (3′-PUA) end which is removed by apurinic endonuclease 1 (APE1). The resulting 3′-hydroxyl group allows DNA polymerase β to fill the nucleotide gap, before being sealed by the DNA ligase III and X-ray repair cross complementing protein 1 (XRCC1) complex (15). As BER is a sequential process where many of the intermediates, such as AP sites or SSBs, are themselves cytotoxic or mutagenic if unresolved (7, 16), flux in the pathway must be tightly regulated to avoid genomic instability (17). However, the AP-lyase step is often rate limiting in bifunctional DNA glycosylases, including in 8-oxoguanine DNA-glycosylase 1 (OGG1) and NTHL1, and the downstream APE1 which is a highly efficient endonuclease can bypass this activity *in vitro* (18, 19, 20, 21, 22), raising questions about the relevance of this function in cells. Moreover, APE1 is generally considered to be ubiquitously expressed in the nucleus so could, in theory, bypass the glycosylase’s AP-lyase activity in cells. However, this has not been thoroughly explored. Although one study found that NTHL1’s AP-lyase activity could still be important in certain sterically occluded nucleosomal DNA damage sites *in vitro* (23), the functional relevance in cells of NTHL1’s AP-lyase activity to overall BER has been debated.

NTHL1 deficiency is implicated in contributing to mutations and genome instability in several human tumor types, for example, the NTHL1 tumor syndrome, showing the importance of this protein (24, 25, 26, 27). However, mice with single knock-out of NTHL1 display no overt phenotype under basal conditions (28). Back-up repair mechanisms make examining the contribution of NTHL1 to BER of oxidative lesions challenging. However, other studies exploring NTHL1 mutant proteins have focused on mutations which exert their functional effect either by not affecting enzymatic activity at all (29), or rendering the enzyme catalytically inactive for both its glycosylase and AP-lyase activities (30, 31, 32). The D239Y catalytically inactive mutant has been reported to exhibit a dominant negative effect, preventing compensatory repair of oxidative DNA lesions, leading to DNA damage and replication stress in mouse embryonic fibroblasts (MEFs) (32). Despite this, the contribution and role of the enzymatic bifunctionality of NTHL1 to its cellular function remains largely unexplored.

In this study, we have generated and characterized a monofunctional-like mutant of NTHL1 which retains glycosylase activity but is deficient in AP-lyase function. Using biochemical and microscopy approaches, we have used this separation of function mutant to determine how disrupting the bifunctional enzymatic activity of NTHL1 impacts its interaction with damaged DNA or chromatin. In cells expressing the monofunctional-like mutant NTHL1 we find increased accumulation of NTHL1 at laser induced DNA damage sites and a decreased protein mobility during oxidative stress, suggesting prolonged engagement of NTHL1 with chromatin. In addition, while more reliant on APE1 for catalytic turnover, the monofunctional-like mutant NTHL1 shows increased competition with APE1 for binding AP sites *in vitro*. In cells, we show that the monofunctional-like NTHL1 mutant, despite showing a small yet increased sensitivity to acute oxidative stress and a change in flux of the BER pathway, likely does not behave in a dominant negative fashion like a catalytically inactive mutant. These findings highlight how a monofunctional-like mutant of NTHL1 with impaired AP-lyase activity can affect chromatin engagement and BER coordination.

## Results

### Characterization of a rationally designed monofunctional NTHL1 mutant *in vitro*

**Figure 1:**
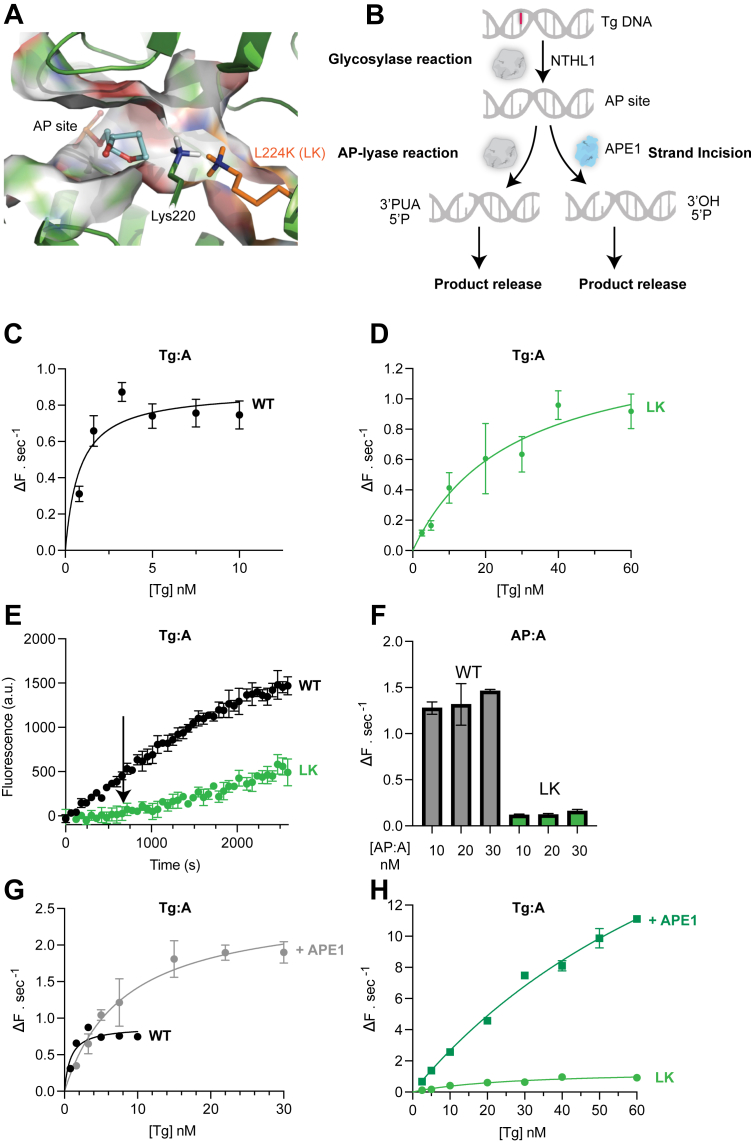
***In vitro* characterization of a rationally designed active site mutant, LK:***A*, model representation of the AlphaFold predicted NTHL1 active site with a rationally designed NTHL1 mutant, L224K (LK), shown in *sticks* representation. The catalytic lysine 220 is indicated. An abasic site from the crystal structure of OGG1 (PDB code 1EBM) is also shown and based on the extensive structural and active site 3D overlap between NTHL1 and OGG1 (see Fig. S1*A*), suggests that the LK lysine is close enough to make potential contacts with substrate DNA in the active site. *B*, schematic of the multistep reaction investigated using an oligonucleotide fluorescence-quencher molecular beacon based assay. NTHL1 first removes thymine glycol (Tg) from the DNA duplex using its glycosylase activity, generating an intermediate apyrimidinic (AP) site. NTHL1 is bifunctional and can further incise the AP site *via* its AP-lyase activity to form a strand break. Alternatively, APE1 can endonucleolytically cleave the AP site to also form a strand break. Release of the strand incised DNA duplex results in fluorescence. *C* and *D*, Michaelis–Menten (MM) plot for WT (*C*) and LK (*D*) of Tg substrate concentration *versus* the initial slope, where the fluorescence increases in a linear fashion, as measured by change in fluorescence per second (ΔF.sec^−1^). Line represents the MM equation fit of the data and error bars indicate the 95% confidence levels of the linear regression line from three technical repeats used to calculate ΔF.sec^−1^. Representative experiment of four independent repeats shown. Enzyme concentration was 10 nM. Parameters extracted from the MM equation fitted data are shown in Table 1. *E*, representative fluorescent curves of WT and LK catalyzed reactions using 5 nM of Tg substrate, with an *arrow* indicating the lagged time point at which a linear increase in product began to be observed in the LK-catalyzed reaction. *F*, LK mutant has diminished AP-lyase activity on a preformed AP site substrate. Initial slopes of reaction of the AP-lyase and product release step on a preformed AP:A substrate at increasing substrate concentrations, with mean and standard deviations from three independent repeats shown. *G* and *H*, representative MM plot for WT + APE1 (*G*) and LK + APE1 (*H*) of initial reaction slopes at increasing concentrations of Tg. *Line* indicates the MM equation fit of the data, and error bars indicate the 95% confidence levels of the linear regression line from three technical repeats used to calculate ΔF.sec^-^1. NTHL1 concentration was 5 nM and APE1 was 2 nM. Data from (*C* and *D*) where NTHL1 alone was tested is shown as a comparison. Representative experiment of three independent repeats shown. The parameters extracted from the MM equation fitted data are shown in Table 2. AP site, apurinic/apyrimidinic site; APE1, AP-endonuclease 1; OGG1, 8-oxoguanine DNA-glycosylase 1; PDB, Protein Data Bank; AP-lyase, apurinic/apyrimidinic-lyase; NTHL1, endonuclease III-like protein 1.

NTHL1 is part of a group of DNA glycosylases possessing both glycosylase and intrinsic AP-lyase activity as opposed to monofunctional DNA glycosylases catalyzing only the glycosylase reaction such as uracil DNA glycosylases UNG or single-strand selective monofunctional uracil DNA glycosylase 1 (SMUG1). Lysine 220 in the active site of NTHL1 is the essential residue for formation of the intermediate Schiff base with the AP site underpinning its importance for the AP-lyase activity (33). However, other bifunctional DNA glycosylases, despite having lysines in their active sites such as OGG1, have been shown to possess an extremely weak AP-lyase activity *in vitro* (18, 19, 20). Considering the fact that APE1 has a much higher activity on AP sites, this raises questions regarding the relevance of the AP-lyase function of OGG1 and other bifunctional DNA glycosylases in cells. It has indeed been shown that the rate-limiting step for NTHL1 is the slow AP-lyase and product release step (21, 22). This indicates that NTHL1’s bifunctionality might be dispensable in cells and might not play a significant role in BER of oxidative DNA base damages. To investigate the importance of its bifunctionality and the intrinsic NTHL1 catalyzed AP-lyase reaction in repairing oxidative DNA base damage, we rationalized an active site mutation which could specifically affect the AP-lyase reaction of NTHL1. We introduced an additional active site lysine by mutating leucine 224 (L224K/LK). We reasoned that this could either increase or decrease the rate of the AP-lyase reaction by providing an alternative lysine for Schiff base chemistry with the substrate in the active site (Fig. 1*A*), without drastic effects on the overall shape of the active site. A similar approach was reported before for the DNA glycosylase MutY, where despite the presence of a lysine in the active site, AP-lyase activity (β-elimination) could only be detected in a S120K mutant (34, 35). Considering the extensive structural fold overlay between NTHL1 and OGG1, including in the active site (Fig. S1*A*), suggesting some commonality in their enzymatic mechanism, we were able to design a NTHL1 mutant in which an introduced lysine could make contact with the substrate inside the active site of NTHL1. In order to characterize the enzymatic activity of NTHL1 LK mutant, we purified the recombinant wild-type (WT) and LK mutant NTHL1 protein (Fig. S2). We expressed a Δ63 amino acid N terminally truncated version of NTHL1 used before *in vitro* (23, 36) (see Experimental procedures) to facilitate its purification, as the disordered N terminus consistently caused the full-length protein to precipitate. The N terminus has been shown to have a role in regulating product release rather than affecting the catalytic step (23, 29, 36, 37). As both WT and LK mutant NTHL1 proteins are truncated, we could use the N terminus truncated proteins to investigate differences in catalytic activity between them. We adapted a fluorescence-quencher (molecular beacon) assay (38) using a defined thymine glycol (Tg) oligonucleotide substrate to assess the activity of NTHL1 under multiple turnover conditions. In the assay, fluorescence is only detected after release of the final product, *i.e.* a DNA strand break, which removes local quenching of the fluorescently labeled DNA strand. This encompasses a multistep reaction for NTHL1, where it must first catalyze the glycosidic hydrolysis to remove Tg and generate an AP site, before forming a DNA strand break using its AP-lyase activity (Fig. 1*B*).

We first attempted to determine the Michaelis–Menten kinetic parameters for the WT catalyzed reaction using multiple turnover conditions on Tg:A substrate. However, we observed significant product inhibition, as reported before (22, 23), most evident at higher substrate concentrations, which precluded us from quantifying true Michaelis–Menten constants. Instead, we determined apparent *K*_M_ and *k*_cat_ values (Fig. 1*C*) for the WT catalyzed reaction, shown in Table 1. However, the LK mutant was found to be saturated at a much higher Tg:A substrate concentration than the WT (Fig. 1*D*) and displayed a 17-fold higher apparent K_M_ compared to NTHL1 WT (Table 1). For a one substrate, single-step enzyme without product inhibition, *K*_M_ can often be an indicator of substrate binding affinity when the substrate binding is part of the rate-limiting step (39). However, owing to the complex multistep nature of the NTHL1 catalyzed reaction, the higher *K*_M_ observed for LK could also be a result from a rate-limiting step in the whole reaction scheme in addition to or instead of solely weaker substrate binding. Moreover, we noted that compared to the WT enzyme there was a substantial time-lag between the reaction start and linear detection of the reaction product in the LK catalyzed reaction (Fig. 1*E*), further suggesting the existence of a different rate-limiting step in the LK mutant catalyzed reaction. To test whether the AP-lyase activity might be the rate-limiting step, we measured the initial rates of NTHL1 WT and LK mutant on a preformed AP site substrate instead of the Tg:A substrate. We observed that the LK mutant displayed a significantly impaired AP-lyase activity compared to the WT enzyme (Fig. 1*F*). Together, these results suggest that the single amino acid substitution in the LK mutant uncouples the glycosylase and AP-lyase activity compared to the WT NTHL1 and transforms NTHL1 into a monofunctional-like DNA glycosylase.

**Table 1:** Apparent Michaelis–Menten constants determined for WT and LK NTHL1 catalyzed reactions

Enzymes	Apparent K_M_ ( ± s.d.) nM	Apparent kcat ( ± s.d.) ΔF.sec^-1^ nM^-1^ (enzyme)	Kcat/K_M_ ( ± s.d.) ΔF.sec^-1^ nM^-1^ (substrate)
Wild-Type (WT)	0.97 ± 0.27	0.063 ± 0.007	0.067 ± 0.012
LK	25.4 ± 2.6	0.10 ± 0.01	0.0040 ± 0.00057

Mean values of parameters extracted from four independent repeats with standard deviations. Enzyme concentration was 15 nM. For the WT enzyme, Tg:A substrate concentrations higher than 10 nM resulted in significant product inhibition so all reported parameters are apparent values. Although similar apparent k_cat_ values for WT and LK were determined, the apparent K_M_ for the LK catalyzed reaction was determined to be approximately 17 fold higher than in the WT. The k_cat_/K_M_ represents the rate constant for the reaction at low substrate concentrations.

The MM equation fit was used to calculate parameters shown in the table.

v = Vmax ∗ [S]/(K_M_ + [S]).

k_cat_ = Vmax/[E]_tot_.

### Active site Leu to Lys mutation results in substantially reduced lyase activity of NTHL1 and stronger dependence on APE1

Under multiple turnover conditions, APE1 increases the formation of product since its efficient endonuclease activity bypasses the AP-lyase step of bifunctional DNA glycosylases such as OGG1 (19, 40), and NTHL1 (22), thereby relieving the rate-limiting step. To further understand whether the LK mutation affects the glycosylase step in addition to the AP-lyase step, we again determined apparent steady state Michaelis–Menten parameters for the WT and LK catalyzed reactions, but with the inclusion of APE1. APE1 has no incision activity on the Tg:A substrate alone (Fig. S1*B*) and was kept in excess (Fig. S1*C*). In addition, we validated that mutating the active site lysine to glutamine (KQ) completely abolished activity, even with the inclusion of APE1 (Fig. S1*B*). We confirmed that APE1 accelerated product formation for the WT and LK catalyzed reactions (Fig. 1, *G* and *H*). Excess APE1 increased the *k*_cat_ of the LK catalyzed reaction almost 10-fold more than for the WT enzyme. This was offset by a 10-fold higher *K*_M_ for the LK catalyzed reaction. Thus, the catalytic efficiency and second order rate constant at low substrate concentrations is similar for the WT and LK enzymes (Table 2). Together, these results demonstrate that the LK mutant behaves more as a monofunctional glycosylase compared to the WT NTHL1, catalyzing the base excision step without being coupled to an AP-lyase reaction. Yet, under sub saturating substrate concentrations in the presence of excess APE1, the two enzymes display similar catalytic efficiencies (*k*_cat_/*K*_M_).

**Table 2:** Apparent Michaelis–Menten constants determined for WT and LK NTHL1 catalyzed reactions with the inclusion of APE1

Enzymes	Apparent K_M_ ( ± s.d.) nM	Apparent kcat ( ± s.d.) ΔF.sec^-1^ nM^-1^ (enzyme)	Kcat/K_M_ ( ± s.d.) ΔF.sec^-1^ nM^-1^ (substrate)
WT + APE1	10.2 ± 1.83	0.695 ± 0.12	0.068 ± 0.017
LK + APE1	102 ± 23.2	6.12 ± 1.35	0.06 ± 0.019

Mean values of parameters extracted from 5 (WT + APE1) or 3 (LK + APE1) independent repeats with standard deviations. NTHL1 concentration was 5 nM and APE1 concentration was 2 nM. A rearrangement of the MM equation was used to calculate apparent K_M_, apparent k_cat_, and k_cat_/K_M_ from the LK + APE1 data. The 10 fold higher k_cat_ determined for LK + APE1 compared to WT + APE1 is accompanied by a 10 fold higher K_M_ of the reaction, thus, under these conditions both enzymes show similar k_cat_/K_m_ values.

**Figure 2:**
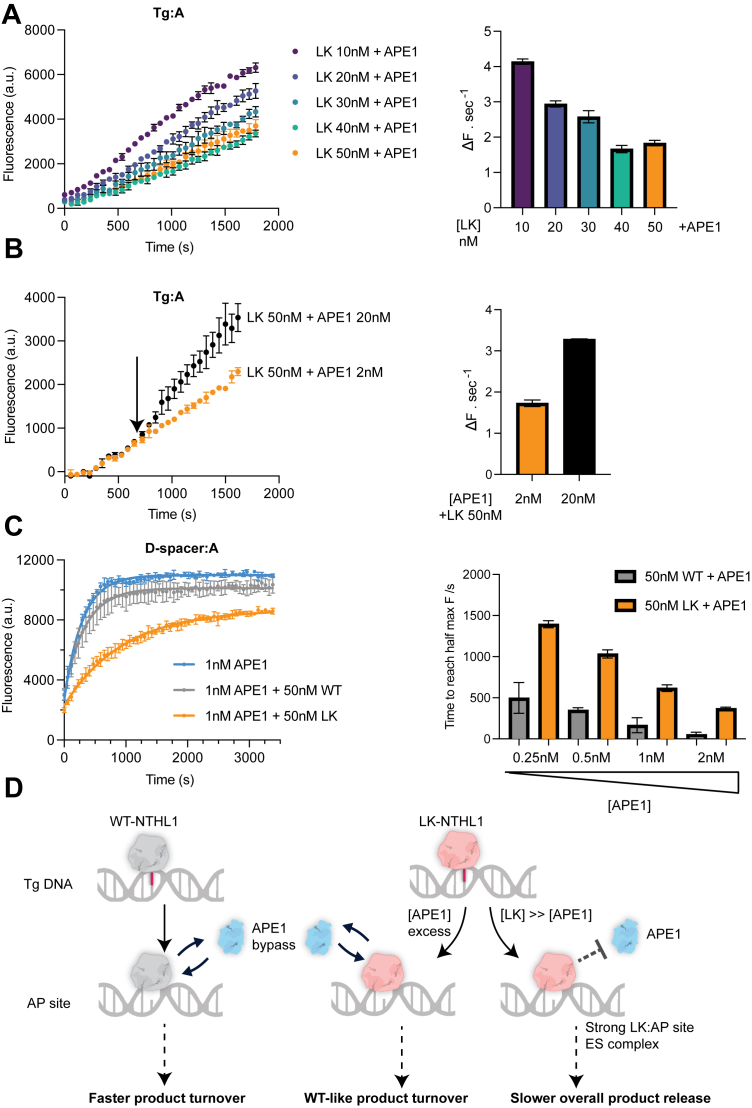
**LK mutant NTHL1 competes with APE1 for binding the intermediate AP site leading to a slower overall product release***A*, representative fluorescent curves of reactions with increasing concentrations of LK enzyme with 2 nM APE1 and 11 nM Tg:A substrate (*left*), showing a general decrease in the initial reaction rate slope as [LK] increases (*right*). Error bars indicate the error of the linear regression from three technical repeats used to calculate the slope. *B*, Subsequently, 20 nM excess of APE1 was added to a reaction containing 50 nM LK and 2 nM APE1 at the indicated time point (*left*), leading to an increase in reaction slope (ΔF.sec^-^1) (*right*). *C*, representative fluorescent curves from an APE1 competition assay (*left*), showing only inclusion of the LK mutant leads to a delay in the time taken to reach maximum fluorescence (*right*). Briefly, 10 nM of a nonhydrolyzable AP site analog (D-spacer) substrate was used, meaning only APE1 cleavage can lead to an increase in product. The time taken to reach half the maximum fluorescence was calculated by fitting data to a one-phase association equation as described in Methods. Experiments were repeated twice and means with standard deviation error bars shown. *D*, model of the different catalytic behaviors of the NTHL1 LK mutant and NTHL1 WT under different conditions *in vitro*. ES, enzyme-substrate complex; AP site, apurinic/apyrimidinic site; APE1, AP-endonuclease 1; Tg, thymine glycol; NTHL1, endonuclease III-like protein 1.

A common feature of monofunctional DNA glycosylases is their apparent tight binding to their product, the AP site. SMUG1, TDG, and AAG are examples of such product-inhibited monofunctional glycosylases (41, 42, 43). We speculated that the LK mutation might convert NTHL1 into a monofunctional glycosylase with tight product binding. To test this, we took advantage of the previously observed drastic differences in AP site cleavage efficiency between the DNA glycosylase and APE1. Increasing the concentration of the LK mutant in the presence of APE1 at the same concentration as used in Figure 1, *G* and *H* led to a slower initial rate of product formation (Fig. 2*A*), a phenomenon not observed with NTHL1 WT (Fig. S1*D*). This suggests that the LK mutant may remain bound at the AP site, thus blocking and delaying access to APE1. To clarify if the LK mutant was trapped either covalently or noncovalently on the AP site, we added excess APE1 to a reaction containing a high concentration of LK mutant and a low concentration of APE1 (Fig. 2*B*). Excess APE1 increased the rate of product release, demonstrating APE1 could compete with the LK mutant for the intermediate AP site (Fig. 2*B*). Thus, the LK mutant may form a tight but noncovalent complex with the intermediate AP site, as opposed to being trapped in a covalent nonproductive complex.

To more precisely compare the activity of the LK mutant and WT NTHL1 at the AP site we performed a competition assay with APE1 using an AP site analog (D-spacer) that can only be cleaved by the endonuclease activity of APE1 (Fig. S3, *A* and *B*). As expected, only inclusion of the LK mutant led to a substantial delay in APE1-mediated cleaving of all available AP site substrate (Fig. 2*C*). This demonstrates that only the LK mutant competes with APE1 for binding to available AP sites while NTHL1 WT does not. Collectively, these results show that the single amino acid substitution in the LK mutant turns NTHL1 functionally into a monofunctional-like DNA glycosylase that is AP site product inhibited *in vitro*. Depending on the context this results in substantial differences in product turnover and repair compared to the bifunctional NTHL1 WT (Fig. 2*D*).

### NTHL1 active site variants display a differential DNA repair response to oxidative DNA damage

**Figure 3:**
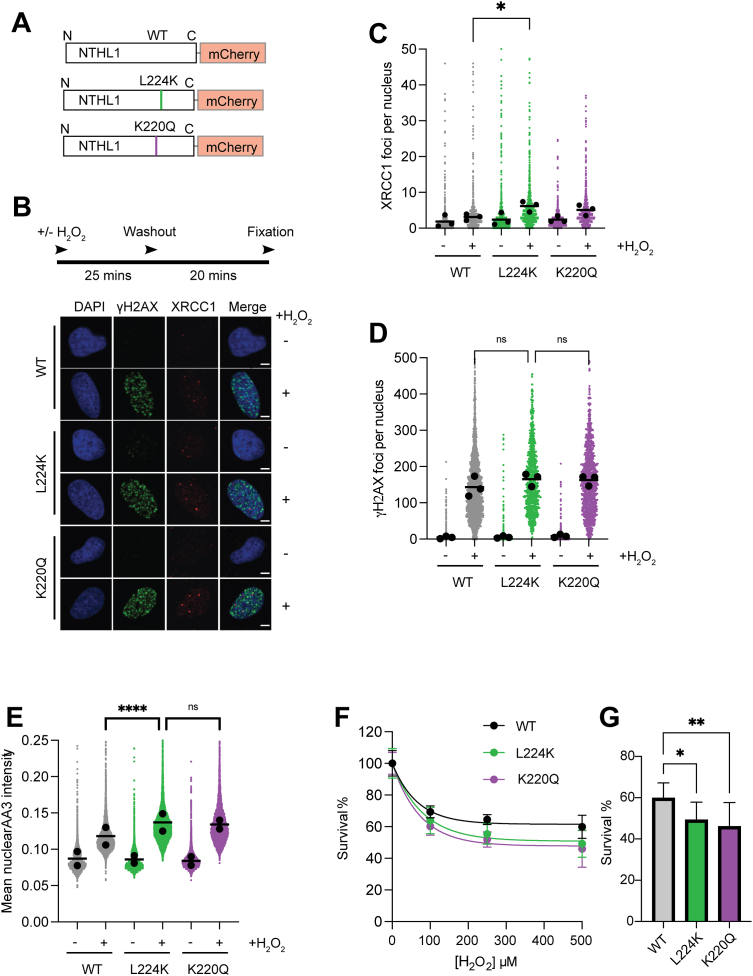
**Generation and characterization of DNA repair and sensitivity to H_2_O_2_ in U2-OS cells expressing WT, monofunctional-like mutant (LK), or catalytically inactive (K220Q) mutant NTHL1.***A*, mCherry C terminal tagged NTHL1 WT, monofunctional-like (L224K), or catalytically inactive mutant NTHL1 (K220Q) were stably reexpressed by transgene overexpression. *B*, experimental outline and representative images of XRCC1 (*C*) or λH2AX (*D*) immunofluorescence of WT, L224K, or K220Q NTHL1 overexpressing cells treated with 500 μM H_2_O_2_ for 25 min, followed by a washout and 20 min recovery period in fresh medium before fixation. The scale bar represents 5 μm. Number of foci per nucleus is shown as a superblot where the means of three independent experiments (*black circles*) are superimposed onto the individual cell data (>2000) (*colored circles*). One-way ANOVA with Sidak’s multiple comparisons test of the mean data (n = 3) was used to calculate significance between groups (*p* < 0.05, ∗∗*p* < 0.01, ∗∗∗*p* < 0.001, ns = nonsignificant). *E*, fluorescence microscopy showing mean nuclear AP site levels in cells cotreated with 1 mM H_2_O_2_ and 10 μM AA3 probe for 1 h. Treated L224K-NTHL1 expressing cells display higher AP site levels compared to WT NTHL1 expressing cells. The mean nuclear intensity of nuclear-bound AA3 probe is displayed as a superblot with means of two independent repeats (*black circles*) superimposed onto individual cell data (>2000) (*colored circles*). Kruskal–Wallis test with Dunn’s multiple comparison test was used to calculate significance between groups (*p* < 0.05, ∗∗*p* < 0.01, ∗∗∗*p* < 0.001, ns = nonsignificant). *F*, decreased survival of L224K and K220Q NTHL1 compared to WT NTHL1 expressing cells upon treatment of increasing doses of H_2_O_2_ for 25 min followed by washout and 24 h recovery period in fresh medium. Survival measured by calculating the percentage of remaining nuclei compared to nontreated cells. Means from three independent experiments performed in triplicate with standard deviations are shown. *G*, bar graph depicting survival percentage at the highest dose of H_2_O_2_ (500 μM) from (*F*). One-way ANOVA with Dunnett’s multiple comparisons test was used to calculate significance between groups (*p* < 0.05, ∗∗*p* < 0.01). AP site, apurinic/apyrimidinic site; XRCC1, X-ray repair cross complementing protein 1; H_2_O_2_, hydrogen peroxide; AA3, O-2-Propynylhydroxylamine hydrochloride; NTHL1, endonuclease III-like protein 1.

Having defined the catalytic properties of WT, monofunctional (LK), and inactive (KQ) NTHL1 with purified proteins, we next asked if the differences in activity translated into differences in the cellular DNA repair of oxidative DNA base damage. To eliminate any competition of endogenous WT NTHL1 in our assays, we first generated NTHL1 knock-out (KO) U2-OS osteosarcoma cells before stably reexpressing either WT, monofunctional (L224K), or catalytic inactive (K220Q) mCherry tagged NTHL1 (Figs. 3*A* and S4). We achieved a similar protein expression level between the different cell lines, but all tagged NTHL1 variants were overexpressed compared to the endogenous NTHL1 level (Fig. S3). We treated cells with hydrogen peroxide (H_2_O_2_) to induce oxidative DNA base damage, including Tg, which is the main lesion recognized by NTHL1. After washout and a repair period we monitored the chromatin retention of downstream BER factor XRCC1 and DNA damage marker λH2AX (Fig. 3*B*). Quantitative confocal microscopy revealed that cells expressing the L224K mutant retained the highest number of detergent resistant XRCC1 foci (Fig. 3*C*), suggesting a slower rate of BER repair. However, the number of λH2AX foci was comparable between all treated cell lines (Fig. 3*D*), suggesting any differences in BER repair did not lead to an immediate transient difference in DNA damage signaling.

We then explored whether the higher number of XRCC1 foci in LK mutant expressing cells might be due to a defect in the BER pathway, such as accumulation of AP sites which are not as efficiently resolved as in cells expressing the WT NTHL1. We used an abasic site probe to specifically quantify nuclear AP site levels in each cell line expressing the different NTHL1 variants (44, 45). AP site levels were significantly higher in the LK mutant compared to the NTHL1 WT expressing cells following H_2_O_2_ treatment, consistent with the monofunctional-like LK mutant generating but inefficiently resolving AP sites (Figs. 3*E* and S5*A*). As the oxidative DNA lesions which NTHL1 processes may generate checkpoint-activating intermediates (46), we asked whether ATR and CHK1 phosphorylation are differentially activated in cells expressing the NTHL1 variants. However, there was no significant difference in the level of CHK1 phosphorylation induced by H_2_O_2_ across the different cell lines (Fig. S6). Nevertheless, a brief, acute H_2_O_2_ treatment revealed a modest yet significant decrease in viability for cells expressing the LK mutant which was comparable to cells expressing the catalytic inactive K220Q mutant and clearly below survival of NTHL1 WT expressing cells (Fig. 3, *F* and *G*). This sensitivity could reflect slower repair of Tg, a potent replicative DNA polymerase blocking lesion. Collectively, although the L224K mutation does not solely affect the AP-lyase activity of NTHL1, these results nevertheless support that a disruption of the bifunctionality of NTHL1 has functional consequences for DNA repair and resistance to oxidative DNA damage in cells.

### Mutation of active site residues within NTHL1 affects its recruitment to DNA damage sites

**Figure 4:**
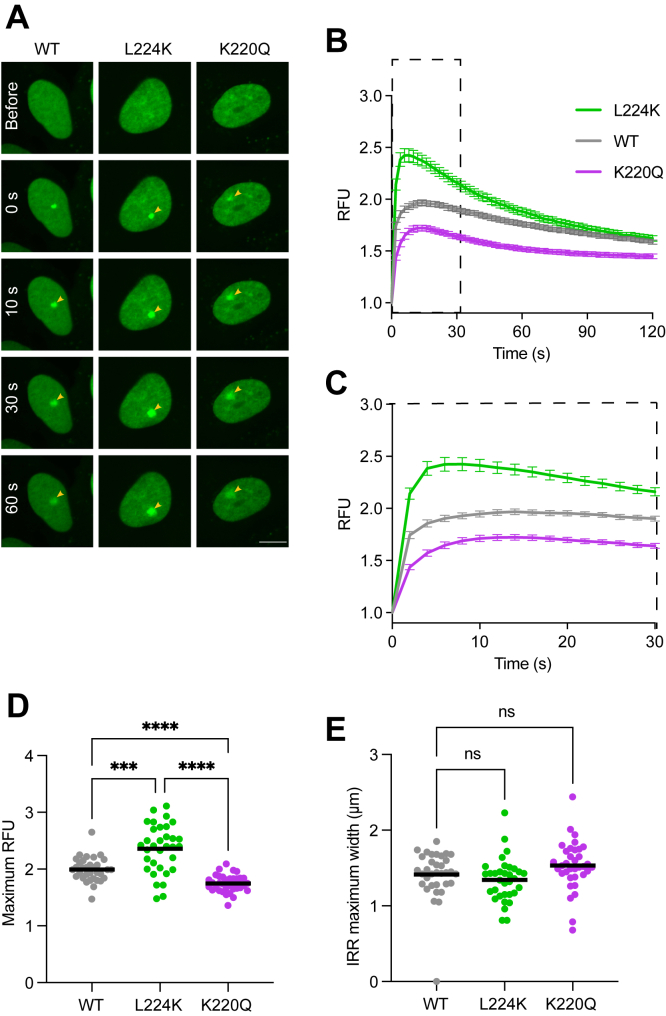
**Mutation of active site residues within NTHL1 affects its recruitment to DNA damage sites.***A*, NTHL1 WT, L224K, or K220Q recruitment kinetics. U2-OS cells expressing either WT, L224K, or K220Q NTHL1-mCherry were presensitized with Hoechst for 10 min and irradiated at a defined subcellular location with a 405 nM laser to induce DNA damage. Representative confocal images from before and after microirradiation, with *arrows* indicating the spot of laser microirradiation. The scale bar represents 5 μm. *B*, quantification of the time course of NTHL1-mCherry recruitment to DNA damage sites described in (*A*). *C*, zoomed in from (*B*) over the first 30 s of acquisition post microirradiation. Data shown as mean and SEM from three independent experiments, >25 cells per condition. *D*, L224K-NTHL1 reaches the highest maximum relative fluorescence intensity (RFU) post microirradiation; however, the width of the recruited spot is unchanged (*E*). Brown–Forsythe’s and Welch’s ANOVA with Dunnet’s multiple comparison test was used to calculate significance between groups (*p* < 0.05, ∗∗*p* < 0.01, ∗∗∗*p* < 0.001, ns = non significant). NTHL1, endonuclease III-like protein 1.

We next evaluated whether active site mutations in NTHL1 might affect its spatiotemporal recruitment to DNA damage sites. We performed laser-induced microirradiation (LIM) of predefined nuclear spots and quantified the recruitment of WT, LK and KQ mutant NTHL1-mCherry to the DNA damage site over time (Fig. 4*A*). All NTHL1 variants displayed rapid recruitment to laser-induced DNA damage sites. However, the monofunctional L224K mutant reached the highest plateau at the damage sites (Fig. 4, *B*–*D*). In contrast, the K220Q catalytic inactive NTHL1 mutant showed the weakest accumulation. The maximum width of the area of accumulation remained unchanged (Fig. 4*E*). Taken together with the previous results, we hypothesize that the L224K mutant likely reaches the highest accumulation as it has higher affinity to and remains bound to the AP site generated by the glycosylase activity or directly by the laser irradiation, more than WT NTHL1. Due to this tighter binding to AP sites, it takes APE1 longer time to displace the mutant glycosylase. The catalytically inactive K220Q mutant may accumulate to a lower level because it can only make brief, reversible contacts with the initial lesions. Lacking catalytic activity, it cannot enter the stage that would cause NTHL1 to accumulate at DNA damage sites. However, due to the mixture of DNA damages induced by LIM, including double-stranded breaks and SSBs and AP sites, in addition to base damage (47), it cannot be ruled out that the L224K may have an altered substrate preference, perhaps preferring to bind AP sites induced by LIM (48) than specific lesions for NTHL1 such as Tg.

### Distinct binding dynamics of NTHL1 active site mutants on oxidative DNA damage

**Figure 5:**
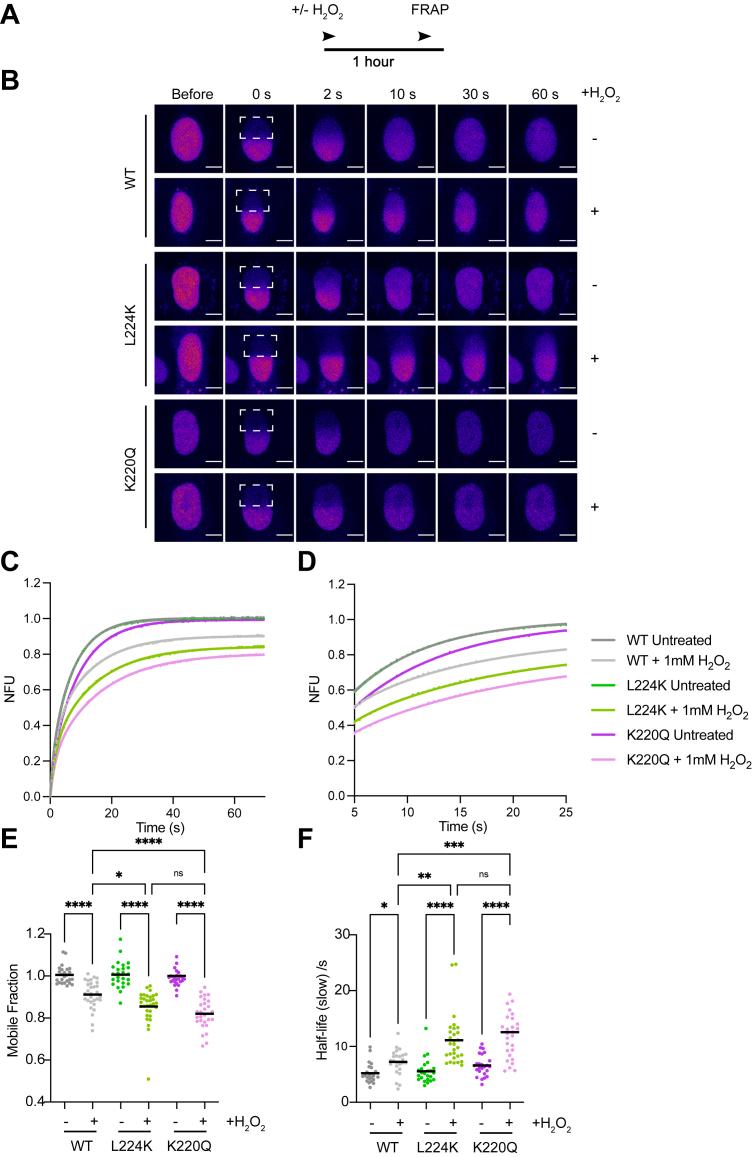
**Distinct *in cellulo* chromatin binding dynamics of NTHL1 mutants exposed to oxidative DNA damage***A*, schematic of experimental setup of fluorescence recovery after photobleaching (FRAP) experiments. *B*, U2-OS cells expressing either WT, L224K, or K220Q NTHL1-mCherry were treated with 1 mM H_2_O_2_ or untreated for 1 h prior to FRAP. Representative confocal images before and after the indicated nuclear region (*white square*) was photobleached, and fluorescence recovery measured over time. The scale bar represents 5 μm. *C*, quantification of NTHL1-mCherry over time in normalized fluorescence intensity units (NFU) after photobleaching. Individual FRAP curves (Fig. S7) were fit to a two-phase association equation (see Experimental procedures), and in (*C* and *D*) is shown the mean and fit from three independent experiments. *D*, zoomed in from (*C*) from 5 to 25 s post bleach. The mobile fraction (*E*) and the half-life of the slow component of the fitted FRAP curves (*F*) are plotted as scatter plots. Data shown are the mean of three independent experiments (n = ≥20 cells) with either standard error (*C* and *D*) or standard deviations (*E* and *F*) error bars. Brown–Forsythe’s and Welch’s ANOVA with Dunnet’s multiple comparison test was used to calculate significance between groups (*p* < 0.05, ∗∗*p* < 0.01, ∗∗∗*p* < 0.001, ns = nonsignificant). H_2_O_2_, hydrogen peroxide; NTHL1, endonuclease III-like protein 1.

If the monofunctional NTHL1 mutant remains bound to its AP site product longer due to a reduced AP-lyase activity compared to the WT enzyme one would expect a difference in chromatin-binding dynamics. To test this, we carried out live-cell fluorescence recovery after photobleaching (FRAP) on oxidatively stressed U2-OS cells expressing mCherry-tagged NTHL1 variants and quantified their nuclear mobility (Fig. 5, *A* and *B*). We fit the resulting FRAP curves (Fig. 5, *C* and *D*) to a two-phase association exponential equation in order to quantify specifically the mobility of the proteins that bind to chromatin and are released, rather than freely diffusing (49, 50, 51). The catalytically inactive NTHL1 K220Q mutant exhibited the slowest recovery, consistent with its ability to bind but not catalyze the removal of oxidized base lesions, therefore only being slowly released from chromatin by spontaneous disassociation (Fig. 5, *D* and *F*). The K220Q mutant also consistently exhibited the lowest mobile fraction after treatment with H_2_O_2_ (Fig. 5*E*). Although WT NTHL1 exhibits the fastest recovery and highest mobile fraction consistent with its rapid catalytic cycle and release of AP site, the NTHL1 L224K mutant exhibits a mobile fraction and half-life more similar to the catalytically inactive mutant (Fig. 5, *D*–*F*). This suggests that the attenuated AP-lyase activity of the NTHL1 L224K mutant results in the glycosylase being chromatin-bound for longer than the WT protein. Thus, the more the NTHL1’s catalytic progression is impeded, the more stably it resides on chromatin: WT NTHL1 is released most quickly after catalysis, L224K NTHL1 remains chromatin bound possibly at the intermediate AP site, and K220Q NTHL1 may be trapped at the lesion recognition stage.

### Post repair FRAP reveals repair completion of WT and L224K NTHL1, but persistent chromatin retention of K220Q NTHL1

**Figure 6:**
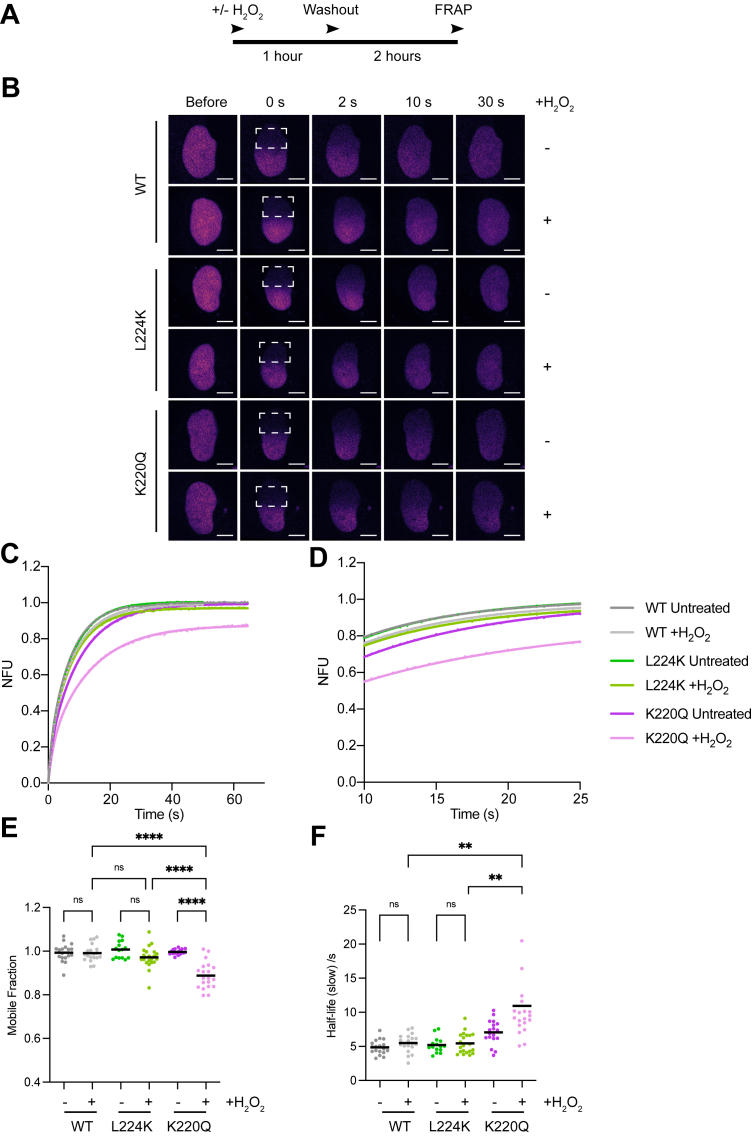
**WT and monofunctional-like (L224K) NTHL1, but not catalytically inactive (K220Q) NTHL1 mutant, recover nuclear mobility after a recovery period***A*, schematic of experimental setup. U2-OS cells expressing WT, L224K, or K220Q NTHL1-mCherry treated with 1 mM H_2_O_2_ or untreated and allowed to recover in fresh media before FRAP. *B*, representative confocal images before and after the indicated nuclear region (*white square*) was photobleached, and fluorescence recovery measured over time. The scale bar represents 5 μm. *C*, mean FRAP curves from the quantification of NTHL1-mCherry recovery (NFU) after photobleaching from three independent experiments. *D*, zoomed in from (*C*) from 10 to 25 s post bleach. Individual FRAP curves (Fig. S8) were fit to a two-phase association equation and the mobile fraction (*E*) and the half-life of the slow component (*F*) plotted as scatter plots. Data shown are the mean of three independent experiments (n = ≥15–21 cells) with either standard error (*C* and *D*) or standard deviations (*E* and *F*) error bars. Brown–Forsythe’s and Welch’s ANOVA with Dunnet’s multiple comparison test was used to calculate significance between groups (*p* < 0.05, ∗∗*p* < 0.01, ∗∗∗*p* < 0.001, ns = nonsignificant). FRAP, fluorescence recovery after photobleaching; H_2_O_2_, hydrogen peroxide; NTHL1, endonuclease III-like protein 1.

Given that the L224K mutant NTHL1 remains chromatin bound for longer than the WT enzyme during oxidative stress, we next asked if this would compromise its ability to complete repair of the remaining DNA lesions once the source of oxidative DNA damage is removed. Cells were treated with H_2_O_2_, then washed out and given a defined recovery period before FRAP was performed (Fig. 6*A*). Mobility measured after the wash out serves as an indirect repair read-out: if NTHL1 moves as freely as in untreated cells, the glycosylase is no longer as stably bound to chromatin and lesions are likely to have been resolved. After the recovery period, the L224K mutant NTHL1 exhibited a half-life of the slow component and mobile fraction indistinguishable from untreated cells (Fig. 6, *B*–*F*), indicating that if given sufficient time, it can finish repair of its oxidative lesions as effectively as WT NTHL1, potentially with help from endogenous APE1. In contrast, in cells expressing the catalytically inactive K220Q NTHL1, lesions are not resolved and the K220Q NTHL1 remains bound at the lesions. This suggests that this mutation acts as a dominant-negative mutation preventing compensatory repair and instead engages in futile chromatin-binding cycles without resolving the oxidative DNA lesions. This is in line with our results in the Tg excision assay showing that APE1 can increase the formation of product with the WT and L224K mutant NTHL1, but not K220Q (Figs. 1, *G* and *H* and S1*B*).

Collectively, our data demonstrate that mutating NTHL1 to be monofunctional-like (L224K) or catalytically inactive (K220Q) causes alterations in both its recruitment to DNA damage sites and its chromatin binding dynamics. Unlike the catalytically inactive mutant NTHL1, the monofunctional-like NTHL1 likely does not act in a dominant negative fashion, because given sufficient time the monofunctional-like NTHL1 mutant recovers its protein mobility in a FRAP assay (Fig. 6, *B*–*F*). However, we show that expression of the monofunctional-like mutant NTHL1 in cells leads to higher levels of BER intermediates and impairs efficient BER completion after acute oxidative stress. Our study illustrates how disrupting the active site of NTHL1 affects the DNA glycosylase’s engagement with chromatin and ultimately the downstream BER.

## Discussion

In this study, we investigated how disrupting the bifunctionality of NTHL1 affects its processing of oxidative DNA base damage in cells. The importance of NTHL1’s AP-lyase activity *in vivo* has not yet been fully determined, as while there exists *in vitro* evidence that it can improve accessibility of sterically hindered sites in nucleosomes for APE1 (23), this has not yet been shown *in vivo*. In addition, several other bifunctional DNA glycosylases utilize APE1 for downstream processing and have weaker AP-lyase activity *in vitro* (22, 52, 53).

Motivated by the question of how altering the balance of NTHL1’s dual activities might affect its cellular function; we investigated the consequences of expressing a monofunctional-like NTHL1 mutant in place of the WT bifunctional enzyme. Mutation of the WT catalytic Lys220, even a conservative substitution, compromises both glycosylase and AP-lyase activity. This is concurrent with previous suggestions that Lys220 (also called Lys212 depending on the start codon considered for translation initiation) is part of a single coordinated catalytic center for both glycosylase and AP-lyase activity (54). Instead, we introduced a single amino acid substitution (L224K) mutation in the active site to affect specifically the AP-lyase step without also impairing the glycosylase activity, which altered the enzyme to be monofunctional-like. Interestingly, a previous study found that an analogous mutation in MutY (S120K), a natively monofunctional DNA glycosylase, allowed the introduced lysine to supplant a different lysine that did not produce beta-elimination activity, and imbued the enzyme with AP-lyase activity, albeit at the expense of overall catalytic efficiency (35). For NTHL1, which has a more robust natural bifunctional activity *in vitro*, we show that substituting an additional lysine (L224K) impairs the AP-lyase activity instead. However, rather than the Lys224 making nonproductive covalent complexes as could be suggested, the introduced lysine appears to instead enhance noncovalent interactions with the AP site, as its binding can still be competed out by excess APE1 (Fig. 2*B*). Therefore, the L224K mutation presumably changes the active site geometry to prevent effective Schiff base complex formation involving Lys220, which is essential for AP-lyase activity.

This product inhibited behavior resembles monofunctional DNA glycosylases such as SMUG1, where it has been suggested that product inhibition acts to protect the resulting AP site (41). NTHL1’s comparatively slower AP-lyase reaction has been suggested to play a similar role to protecting the AP site by keeping the AP site in a covalent complex for longer with the DNA glycosylase. This highlights the different ways a bifunctional glycosylase could exert a regulation over the levels of unprotected AP sites.

We find that cells expressing the monofunctional-like mutant NTHL1 accumulate more AP sites after oxidative stress, possibly due to increased competition with APE1 for the AP site. Downstream BER is affected because we also observe a higher persistence of XRCC1 foci. This is consistent with a decoupling between the rate of AP site production and subsequent resolution, which indicates the monofunctional mutant contributes to a reduced overall BER of oxidative lesions, with a rate-limiting step of the overall BER close to the rate of AP site resolution. This could cause a slow hand-off to the downstream enzymes in BER. Despite this, we detected no significant difference in DNA damage signaling between cell lines expressing the various NTHL1 variants, either under basal conditions or after acute oxidative stress. This was unexpected given that clustered oxidative lesions can, during attempted BER, give rise to DNA damage such as DNA double-strand breaks when opposing strands are simultaneously processed (55, 56, 57).

A previous report demonstrated that overexpression of NTHL1 induces DNA damage and genomic instability in nontransformed cells (58). In contrast, our study was performed in U2-OS osteosarcoma cells, where compensatory mechanisms, potentially linked to their high speed of replication, may mitigate instability caused by NTHL1 overexpression. Notably, U2-OS cells retain functional p53 (59), which has been shown to stimulate NTHL1 activity during oxidative stress (60), supporting the relevance of this model for investigating the role of NTHL1 bifunctionality in cellular responses to oxidative damage.

Further implicating a role for the AP-lyase function at accumulation of DNA damage sites *in vivo*, we found that the monofunctional-like NTHL1 mutant accumulated more strongly at LIM DNA damage sites than the WT NTHL1. This is consistent with prolonged retention at AP sites and slower hand-off to downstream BER enzymes. Conversely, the catalytically inactive mutant (K220Q) NTHL1 showed a weaker accumulation than WT NTHL1, showing a difference to a recent report where it showed a slower but eventually higher accumulation after proton beam induced DNA damage. These differences in accumulation might be due to the differences in relative composition of the induced DNA damages between proton beam and LIM, as well as other specific conditions such as the cell type and expression level of NTHL1 used.

Finally, we showed that the bifunctionality of NTHL1 impacts its chromatin association; the monofunctional-like mutant NTHL1 is chromatin bound for longer due to delayed turnover. This makes it more similar to the catalytically inactive mutant than WT NTHL1 during oxidative stress. Notably, unlike the catalytically inactive mutant, its mobility was restored after a defined repair period suggesting repair can be completed given enough time and APE1 assistance. Despite this distinction in repair capability, both monofunctional-like and catalytically inactive NTHL1 mutants display a similarly increased sensitivity to acute H_2_O_2_ treatment, suggesting another factor such as increased chromatin retention time may play a role in the pathogenicity of the NTHL1 mutants to oxidative stress.

Prolonged chromatin association of DNA glycosylases could have implications beyond direct lesion processing, potentially interfering with transcription or replication progression, or even have epigenetic consequences (61, 62, 63). For example, it was recently reported that the OGG1 S326C variant induces a prolonged transcriptional proinflammatory response due to its extended association with DNA (64). Although our study did not assess the exact mechanism by which expression of monofunctional-like NTHL1 mutant leads to a survival defect after acute oxidative stress, it is in line with a previous report where overexpression of WT NTHL1 enhanced cellular survival to H_2_O_2_ and knock-down reduced survival (55). A possible explanation for the observed survival defect is that increased levels of unprotected AP sites can give rise to DNA strand breaks during replication. Alternatively, our findings raise the possibility that another factor contributes to the phenotype observed, beyond the more well-established role of unrepaired Tg lesions in stalling replicative polymerases due to expression of dominant negative catalytically inactive NTHL1 (32). The increased chromatin association of glycosylases such as monofunctional-like NTHL1, which do not act in a dominant negative fashion, may interfere with transcription or replication through collisions with polymerases, a mechanism that may play a more significant role in genome instability than previously appreciated. Supporting this, prior single molecule studies on a catalytically inactive OGG1 mutant found that it remained bound to 8-oxo-G 23-fold longer than the WT (65) and potentially displayed some nonspecific DNA binding (66), a feature that may be shared with monofunctional-like NTHL1 mutant and merits future investigation. Overall, our findings demonstrate that disrupting the bifunctionality of NTHL1 not only impairs enzymatic activity but also alters repair timing, chromatin retention dynamics, and cellular resilience to oxidative stress.

## Experimental procedures

### NTHL1 activity assay

#### Thymine glycol activity assay

Tg substrate was a 22-mer oligonucleotide duplex, created by annealing a fluorescently labeled and Tg containing strand to its complementary quencher-containing strand at a ratio of 1:1.25. The sequences of the oligos are listed in Table S1. The protocol was adapted from (38), with some modifications. Briefly, the assay was performed in black 384-well plates (OptiPlate, #6007279 PerkinElmer) using final concentrations of 25 mM Tris–HCl pH 8.0, 15 mM NaCl, 2 mM MgCl_2_, 0.5 mM DTT, and 1:3000 dilution of dialyzed fish gelatin (Sigma-Aldrich G7765). Ten microliters of NTHL1 enzyme solution for a final concentration of 5 nM, 15 nM, or 50 nM as indicated, was added to wells before addition of 40 μl of substrate in order to start the reaction. Plate was very briefly spun to mix assay reagents and fluorescence was measured every 60 s using a Hidex plate reader at 25 °C (serial no. 3140045; version v1.3). Fluorescence from carboxyfluorescein was measured using an excitation 485 nm filter with bandwidth of 10 nm, and a 535 nm emission filter with a bandwidth of 20 nm. Unless otherwise specified in the figure legends, background fluorescence from the equivalent concentration of DNA substrate alone was subtracted prior to analysis of the fluorescence curves. Three technical repeats were recorded for each condition.

#### Abasic site activity assay

The method was identical to the Tg activity assay except for the substrate used. AP site substrate was freshly generated through incubation of annealed U:A duplex (Table S1) with 5 nM hUNG2 protein for 30 min at 25 °C before use in the assay.

#### D-spacer AP site competition assay

The method was identical to the Tg activity assay except D-spacer AP site containing duplex was used as the substrate (Table S1). Data were fit to a one-phase association exponential equation in GraphPad Prism v10.

Y=Y0+(Plateau-Y0)∗(1-exp(-K∗x))

#### Michaelis–Menten kinetic analysis

Data from the Tg activity assay were used to determine apparent Michaelis–Menten kinetic parameters for the NTHL1 WT and LK mutant catalyzed reactions. The initial rates at different substrate concentrations (shown on the x axis of Fig. 1, *C*, *D*, *G*, and *H*) were plotted against substrate concentration, and the Michaelis–Menten equation was fit to the data using nonlinear regression in GraphPad Prism v10.

#### CRISPR Cas9 KO cells

NTHL1 CRISPR-Cas9 KO U2-OS cell line was generated through transfection of TrueCut Cas9 Protein v2 (Thermo Fisher Scientific, #A36498) and a pool of guide RNAs targeting exon 2 of NTHL1 (Gene Knockout Kit v2, Synthego) with TransIT-X2 transfection reagent (Mirus, #MIR6004) according to manufacturer’s instructions. After 48 h, cells were subcloned by dilution into 96-well plates and grown to confluency. All single KO clones were validated both by Western Blot and Sanger sequencing of the genomic region containing the fragment deletion and/or indels resulting from CRISPR-Cas9. The ICE tool (Synthego) was used to validate the knock-out at the DNA sequence level (https://ice.editco.bio/#/). Guide RNAs (gRNAs) used are listed in Table S2, and primers used are listed in Table S3.

#### Construction of WT, L224K, and K220Q NTHL1-mCherry plasmids

To generate mCherry tagged NTHL1 overexpression vectors, the human NTHL1 coding sequence excluding the stop codon was cloned from the pRetroQ-NTHL1-mCherry plasmid (47) using the NheI-BamHI restriction sites and ligated into the constitutive mammalian expression vector mCherry2-N1. mCherry2-N1 was a gift from Michael Davidson (Addgene plasmid # 54517; http://n2t.net/addgene:54517; RRID:Addgene_54517). Plasmids were purified using GeneJET plasmid miniprep kit (Thermo Fisher Scientific, #K0503) following manufacturer’s instructions. Correct sequences were verified by direct sequencing and plasmids were used for transfections or as template for generating mutant expression constructs of NTHL1. Site-directed mutagenesis was carried out by whole-plasmid PCR with custom primers (listed in Table S3) using Phusion High Fidelity Polymerase Mix (Thermo Fisher Scientific, #F531). After overnight digestion with DpnI (Thermo Fisher Scientific, #FD1703) PCR product was transformed into *E. coli* TOP10. Plasmids were purified and correct sequence was verified using sequencing. Successfully mutated plasmids were purified by miniprep and used for transfections.

#### Generation of stable cell lines expressing WT, L224K, or K220Q NTHL1-mCherry

WT, L224K, or K220Q NTHL1-mCherry plasmids were transfected into the generated NTHL1 KO cells using jetPEI (Polyplus, #101000020) according to manufacturer’s instructions, and after 48 h was selected with 1 mg/ml G418 for 7 days.

#### Cell culture and treatments

U2-OS cells were purchased from European Collection of Authenticated Cell Cultures (#92022711). U2-OS cells were cultured at 37 °C in a 5% CO_2_ atmosphere in Dulbecco’s modified Eagle’s medium (DMEM) + GlutaMax (Gibco) supplemented with 10% fetal bovine serum (FBS) (Gibco) and 100 U/ml penicillin–streptomycin (Gibco). Cells were routinely checked for *mycoplasma* contamination using Lonza *Mycoplasma* Detection Kit (Lonza, LT07–318). Hydrogen peroxide (Thermo Fisher Scientific, #L14000-AP) diluted into DMEM was used to induce oxidative DNA damage.

#### Western blot

Cell pellets were lysed on ice for 30 min in NP-40 lysis buffer (150 mM NaCl, 1 mM EDTA, 50 mM Tris–HCl, and 1% NP-40) supplemented with protease inhibitor (Roche, #04693159001) with intermittent vigorous vortexing. Lysates were clarified by centrifugation at 13,000*g* for 30 min at 4 °C and the supernatant frozen at −80 °C. Bicinchoninic acid (BCA) assay was used to determine protein concentration (Thermo Fisher Scientific, #23225), and 10 μg of protein mixed with 4× NuPAGE LDS sample buffer (Invitrogen, #NP0007) and 10× NuPAGE sample reducing agent (Invitrogen, #NP0009). Electrophoresis was carried out using 4 to 15% Mini-PROTEAN TGX gel (Bio-Rad, #4561083) and proteins were blotted onto nitrocellulose membranes using Trans-Blot turbo transfer system (Bio-Rad, #1704150). Membranes were blocked in 5% nonfat dry milk in tris-buffered saline with Tween (TBST) and incubated with primary antibodies (listed in Table S4) overnight at 4 °C. After 3 × 5 min washes in TBST, secondary antibodies (see Table S4) diluted in 5% milk were incubated with membranes for 1 h at room temperature protected from light. After 5 × 5 min washes in TBST, membranes were visualized using an Odyssey CLx Infrared Imaging system (LI-COR). ImageStudioLite v5.2 (LI-COR) was used for image analysis, and ImageJ was used for quantification of bands.

#### Immunofluorescence

Cells grown on ibidi 8-well with #1.5 polymer (180 μm) coverslip bottom (Ibidi, #80826) were incubated in ice-cold pre-extraction buffer (10 mM Pipes pH 6.8, 100 mM NaCl, 300 mM sucrose, 1 mM EDTA, and 0.5% Triton X-100) for 5 min on ice, washed with phosphate buffered saline (PBS), and fixed in 4% paraformaldehyde in PBS for 10 min at room temperature. After 3 × washes in PBS, permeabilization buffer (0.5% Triton X-100 in PBS) was added for 5 min, before a further wash and incubation with blocking solution of 4% bovine serum albumin (Sigma-Aldrich, #A9418) in PBS for 1 h at room temperature. Cells were then incubated with primary antibody (see Table S4) in 2% bovine serum albumin in PBS overnight at 4 °C. After 3 × 5 min washes in PBS, the appropriate secondary antibodies conjugated to fluorophores were incubated for 1 h at room temperature. After 5 × 5 min washes in PBS, cell nuclei were stained with 4′,6-diamidino-2-phenylindole (DAPI, 5 μg/ml, MilliporeSigma, #D9542). Images were acquired on a Nikon Ti2 inverted confocal microscope (Nikon AX) using a Plan Apo λ D 20 × /0.75 NA air objective. Single-plane images (1024 × 1024 pixel, 12 bit) were acquired in Galvano bidirectional scanning mode with 4 × line averaging and a pixel dwell time of 0.3 μs, with pinhole at ≈ 1 AU. Fluorophores were excited sequentially with 405 nm (3%), 488 nm (0.5%), and 640 nm (6%) laser lines, and emission collected at 429 to 474 nm, 503 to 541 nm, and 662 to 737 nm, respectively. Minimal-crosstalk channel series and line-series acquisition were used to avoid bleed-through. At least 65 images per condition were captured using a custom JOBS module in NIS-Elements AR v6.10.01. Images were further analyzed using CellProfiler software v4.2.6, where number of XRCC1 and λH2AX foci were quantified per nuclei, which was segmented using the DAPI channel.

#### Quantitative AP site microscopy assay

Cells cultivated in PhenoPlate 96 well-plates (Revvity, #6055302) were incubated with 10 μM O-2-propynylhydroxylamine hydrochloride (AA3) probe (Thermo Fisher Scientific, # 438810010) and diluted in DMEM for 1 h. To prepare cells for staining, unbound probe was removed by incubating cells with pre-extraction buffer (mentioned above) supplemented with 1 μg/ml RNase A (Thermo Fisher Scientific, #EN0531) for 5 min on ice. Cells were then fixed and permeabilized with methanol:acetone (1:1) for 5 min on ice. After 3× PBS washes, AA3-bound DNA was labeled with Alexa Fluor 647 (AF647) azide using click chemistry. A solution containing 4 mM copper(II)-sulfate, 2 μM AF647 azide (Jena Bioscience, #CLK-1299), and 10 mM ascorbate in PBS was added to cells for 30 min at room temperature. After 5 × 5 min PBS washes, nuclei were labeled with 5 μg/ml DAPI in PBS for 5 min. After a final wash, images were acquired using a Tecan Spark Cyto plate imager using an Olympus 10×/0.30 NA air objective in the fluorescence imaging module. Fluorophores were excited sequentially using a LED light source, with DAPI excited at 381-400 nm at LED intensity 70%, and emission detected at 445 nm with a 60 nm bandwidth filter after 30 ms exposure. AF647 was excited at 626-644 nm at LED intensity 100%, and emission detected at 685 nm with a 40 nm bandwidth filter after 800 ms exposure. A sCMOS camera (2048 x 2048 pixel) was used. Images were saved as 12 bit TIFF files *via* SparkControl v4.0 software and analyzed using CellProfiler v4.2.6. The DAPI channel was used to segment nuclei, and the mean fluorescent intensity of AF647 quantified.

#### Nuclei counting/viability assay

Three thousand cells per well were seeded into a 96 well-plate (Corning, #3595) and allowed to adhere overnight. After hydrogen peroxide treatment (0–500 μM) for 25 min, the media were removed and replaced with fresh media to allow the cells to recover. After 24 h, the media were removed to remove dead cells, and remaining cells were fixed in 4% paraformaldehyde and stained with cell permeable 1 mg/ml Hoechst 3342. Images were acquired on a Nikon Ti2 inverted confocal microscope (Nikon AX) using a Plan Apo λ D 4X/0.20 NA air objective. Single-plane images (2048 x 2048 pixels) of Hoechst fluorescence, excited at 405 nm and emission at 561 nm, were captured using NIS-Elements AR v6.10.01 software. CellProfiler v4.2.6 was used to segment and count the number of nuclei per well, and survival was calculated by calculating the percentage of nuclei in samples with treated compared to untreated cells.

#### Microirradiation and live-cell microscopy

U2-OS cells expressing either WT, L224K, or K220Q NTHL1-mCherry were seeded in μ-Dish_35mm_ Grid-500 (Ibidi, #81166) and left to attach overnight. Cells were presensitized with 10 μg/ml cell permeable Hoechst 3342 for 10 min at 37 °C before replacement of the media with temperature and pH preequilibrated live cell imaging media (Thermo Fisher Scientific, #31053028) supplemented with 10% fetal bovine serum, 1 mM sodium pyruvate, and 25 mM Hepes. Live cell imaging was performed in a Zeiss LSM780 confocal microscope with a temperature and CO_2_ adjustable (37 °C, 5% CO_2_) chamber on an Axio Observer Z1 inverted stand, and a UV-transmitting Plan-Apochromat 40 × /1.30 Oil DIC M27 objective. To perform microirradiation, a nuclear spot of 10-pixel diameter was selected for microirradiation with the circular region tool (ZEN, Zeiss). DNA damage was induced using a 405 nm diode laser (intensity 100%, zoom 10, pixel dwell time 50 μs, 1 iteration), and images acquired every 2 s afterward. mCherry fluorescence was excited at 561 nm DPSS laser (2% laser power) and emission from 570-694 nM detected with GaAsp spectral detector (512 × 512 pixel size, line averaging 2, ≈1.5 AU pinhole). Image stacks were saved as .czi files and loaded into Fiji v2.1.0 for analysis. For quantification of NTHL1 recruitment to microirradiation, a recursive alignment of the stacked images was performed using plugin StackReg to compensate for slight movement of cells in x-y (67). The fluorescence intensity of a 30-pixel diameter circle covering the irradiated area was quantified, and background fluorescence accounted for by subtracting the background fluorescence of the whole nucleus and a background area. The average intensities from six images acquired preirradiation were set as 1, and all subsequent measurements were normalized to this value. The maximum diameter of the recruited spot was measured in Fiji v2.1.0.

#### Fluorescence recovery after photobleaching

Cells were seeded in 4-well μ-Dish 35 mm (Ibidi, #80466), and after overnight attachment, exposed to 1 mM hydrogen peroxide diluted in live cell imaging medium for 1 h. FRAP was performed in a similar method to (38) with some changes. A Zeiss LSM780 confocal microscope with a temperature and CO_2_ adjustable chamber (37 °C, 5% CO_2_) on an Axio Observer Z1 inverted stand, and a UV-transmitting Plan-Apochromat 40 × /1.30 Oil DIC M27 objective was used. Half of the nucleus was selected using regions tool (Zen software, Zeiss) and photobleached with 561 nm DPSS laser line (100% laser power, 6.3 μs pixel dwell time, 10 frame-scan iterations). Bleaching settings were validated prior using fixed samples and confirmed they did not generate long-lived dark states. An image series were acquired every 500 ms, (20 prebleach and 130 post bleach), with a frame size of 256 × 256 pixels and a pixel size of 83 nm, bidirectional scanning with no line averaging, at a pinhole setting of 2.17 AU. mCherry fluorescence was detected from 579-694 nM with the 32-channel GaAsP spectral detector. Nuclei of very similar size and fluorescence intensity were selected for FRAP analysis to minimize the effect of this variability on FRAP results.

For analysis, the image stacks were loaded into Fiji v2.1.0 and the mean fluorescent intensities of the bleached region, the nucleus, and background were measured. The fluorescent intensity of the bleached region was corrected for the background intensity and overall loss of fluorescence in the nucleus over time. The mean intensities from the first 20 images (prebleach) were averaged, set to 1, and the first post bleach intensity set to 0. The data were visualized in GraphPad Prism v10 and fit to a two-phase exponential association equation. The PercentFast determines the contribution of the faster component to the final fit. In all experiments, this contribution was < 50%.

SpanFast=(Plateau-Y0)·PercentFast·0.01

SpanSlow=(Plateau-Y0)·(100-PercentFast)·0.01

Y=Y0+SpanFast·(1-e-KFast·x)+Spanslow·(1-e-KSlow·x)

#### Cloning of recombinant expression vectors

For recombinant expression, we used a human NTHL1 construct that lacks the first 63 N terminal residues, as previously described (23, 36). NTHL1 constructs were assembled into prelinearised (Bsal-HFv2 restriction enzyme) pNIC 28-Bsa4 (gift from Dr Opher Gileadi) expression vector using Gibson assembly. Gibson assembly was carried out using NEBuilder HiFi DNA Assembly Master Mix (New England Biolabs). The assembly reaction product was transformed to NEB5α (gift from Dr Opher Gileadi) and cells plated onto LB agar plates supplemented with 50 μl/ml kanamycin and 5% sucrose. The success of assembly was confirmed by whole-plasmid sequencing.

#### Protein expression and purification

NTHL1 proteins were expressed following the standard Structural Genomics Consortium protocol (68, 69). Briefly, proteins were overexpressed from the pNIC 28-Bsa4 vector in *E. coli* BL21(DE3)-R3-pRARE2 cells. Cells were grown in Terrific broth (TB) media supplemented with 50 μl/ml kanamycin and 34 μl/ml chloramphenicol. Autoinduction was performed by addition of 0.1 mM isopropyl β-d-1-thiogalactopyranoside at 18 °C overnight. Cells were lysed by sonication on ice in 50 mM Hepes pH 7.5, 0.5 M NaCl, 10 mM imidazole, 10% (v/v) glycerol, 0.5 mM Tris(2-carboxyethyl)phosphine hydrochloride (TCEP), with 1 ml/g protease inhibitor (Complete EDTA-free tablets, Roche). Cell lysate was cleared at 22,000 rpm for 30 min, passed through a 0.22 μm filter (Starlab), and then passed over an AKTAxpress and HisTrap excel column (Cytiva). Proteins were eluted by pooling fractions in 50 mM Hepes pH 7.5, 0.5 M NaCl, 300 mM imidazole, 10% (v/v) glycerol, and 0.5 mM TCEP. After an overnight incubation at 4 °C with tobacco etch virus (TEV) protease, dialyzed proteins were further purified (Ni-binding proteins and TEV protease removed) using a reverse purification with HisTrap excel, 1 x 1 ml column (Cytiva) in 10 mM Hepes pH 7.5, 0.3 M NaCl, 5% (v/v) glycerol, and 0.5 mM TCEP. The protease cleavage of the 6xHis tag was verified using SDS polyacrylamide gel electrophoresis (SDS-PAGE) as described in SDS-PAGE. Proteins were concentrated using a 10K MWCO centrifugal concentrator (Thermo Fisher Scientific), aliquoted, flash frozen in liquid nitrogen, and stored at −80 °C in 10 mM Hepes pH 7.5, 0.3 M NaCl, 20% (v/v) glycerol, and 0.5 mM TCEP. Purified APE1 protein was used from a purification which is described and shown in (38).

#### SDS-PAGE

Protein samples were mixed with 4× NuPAGE LDS Sample Buffer (Invitrogen) and denatured at 95 °C for 10 min. The samples alongside PageRuler Unstained Broad Range Protein Ladder (Thermo Fisher Scientific) or PageRuler Plus Prestained Protein Ladder (Thermo Fisher Scientific) were loaded onto NuPAGE 4 to 12% Bis-Tris Gel. The gels were exposed for 60 min at 150 V in NuPAGE MOPS SDS Running Buffer (Invitrogen) and were stained using Coomasie SimplyBlue SafeStain (Invitrogen).

#### Mass spectrometry analysis

Mass spectrometry was performed to confirm the purity of the purified proteins. The mass spectrometry analysis was done at the ANA Futura Mass Spectrometry Facility at Karolinska Institutet by Dr Mats Johansson. The analysis was done on a UPLC XEVO G2 XS high resolution LC/MS system from Waters. In addition, 5 μl of the protein sample was injected on a BioResolve RP mAb Polyphenyl, 450 Å, 2.7 μm column using a short gradient of MQ water and acetonitrile, both with 0.1% difluoroacetic acid. Mass spectrometry detection was done, acquiring from 500 to 3000 m/z, and the multiple charged cluster was deconvoluted with MaxEnt1 software to obtain the neutral mass of the intact protein.

## Statistical analysis

Statistical methods used are detailed in each figure legend. Normality tests such as Kolmogorov–Smirnov and visual inspection of the associated QQ plot was used to test normality for each analysis. The appropriate analyses were performed in GraphPad Prism v10.

## Data availability

The authors declare that the data supporting the findings of this study are available within the article and its supporting information files. Should any raw data files be needed in another format they are available from the corresponding author upon reasonable request.

## Supporting information

This article contains supporting information.

## Declaration of generative AI and AI-assisted technologies in the writing process

ChatGPT4 was used to edit sentences and sentence structure for improved clarity of the manuscript. The authors reviewed the outputs of AI tools, edited them as necessary, and take full responsibility for the content of the article.

## Conflict of interest

The authors declare that they have no conflicts of interest with the contents of this article.
