# Supplementary material for: A monofunctional-like mutant of DNA glycosylase NTHL1 changes the dynamics of DNA repair during acute oxidative stress
Source: J Biol Chem. 2026 Feb 27;302(4):111332. doi: 10.1016/j.jbc.2026.111332 (PMC13022620; doi:10.1016/j.jbc.2026.111332)
Supplement: Supplementary Figures and Tables [file mmc1.pdf]

A monofunctional-like mutant of DNA glycosylase NTHL1 changes the dynamics of DNA repair during acute oxidative stress

James Haslam, Natalie Rudolfova, Kaixin Zhou, Evert Homan, Ann-Sofie Jemth, Maurice Michel, Thomas Helleday, Oliver Mortusewicz

Supplementary Information includes:

Supplementary Tables 1-4

Supplementary Figures 1-8

Supplementary Figure 1

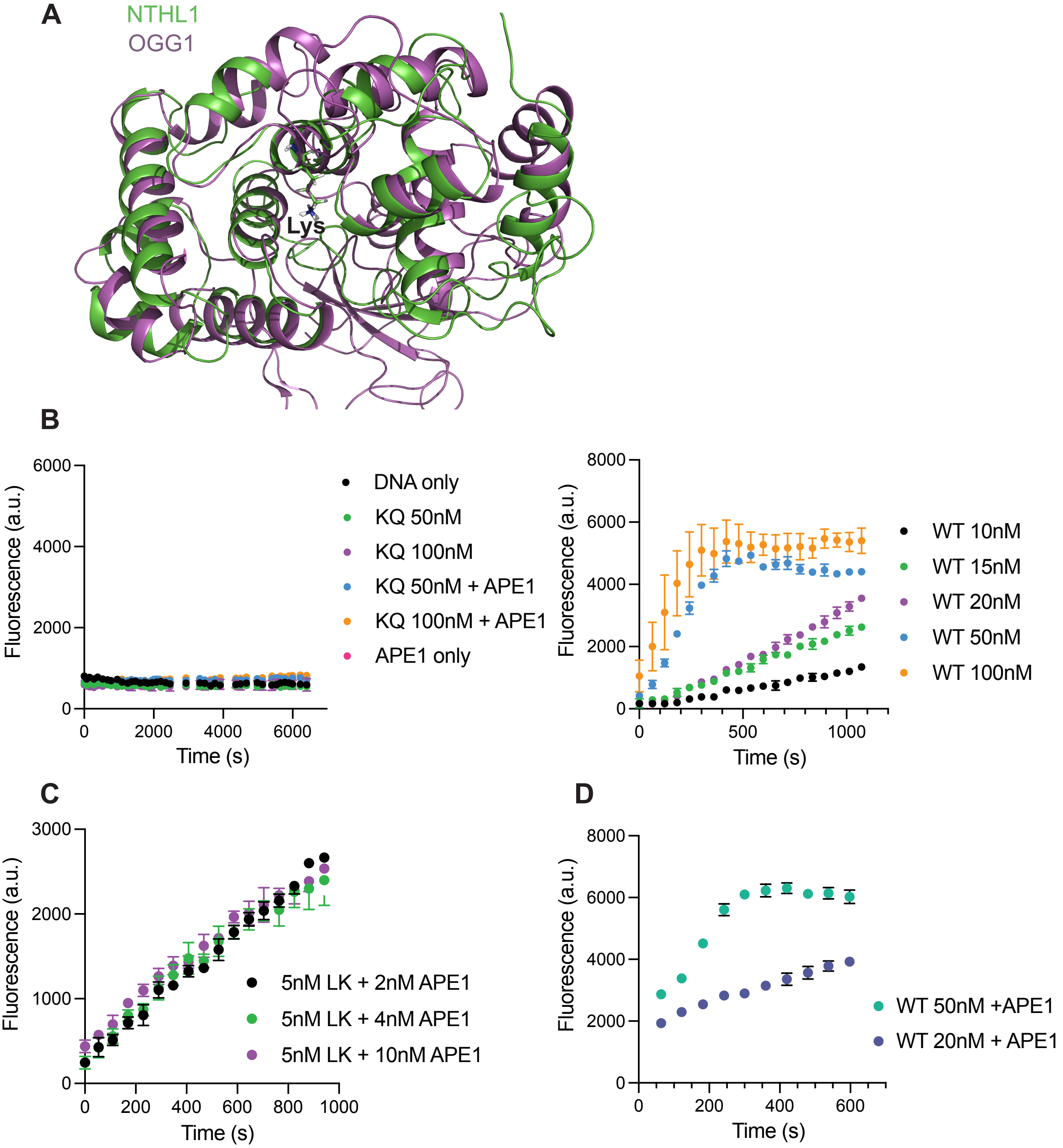

Supplementary Figure 1: NTHL1 requires the conserved catalytic lysine, which has structural homology with OGG1, for activity on a Tg:A containing DNA substrate A. Representation of the extensive structural fold overlap between OGG1 (purple) (PDB code 1EBM) and NTHL1 (green) AlphaFold3's predicted model. Shown in sticks representation is the superimposition of the catalytic lysines (Lys) from OGG1 (Lys249) and NTHL1 (Lys220), essential for AP-lyase activity via Schiff base formation with the abasic sugar, underscoring their shared bifunctional enzymatic mechanism. Structures superimposed by least-squares alignment of alpha-carbon backbone atoms in Pymol. **B.** Representative fluorescence curves (left) showing mutation of the catalytic lysine 220 to glutamine (K220Q) eliminates any detectable activity on a Tg substrate above background (DNA only), even with APE1 (2nM) present. APE1 alone cannot catalyse incision of the DNA backbone without a DNA glycosylase present to first catalyse the glycosylase reaction on a Tg substrate (10nM). Different concentrations of WT NTHL1 showing an increase in initial reaction rate at increasing enzyme concentrations from the same experiment is shown as a comparison (right). Graphs shown are representative of 3 independent repeated experiments. **C.** Representative fluorescence curves of LK (5nM) with increasing concentrations of APE1 showing no further increase in initial reaction rate on a Tg substrate (11nM), indicating 2nM APE1 is already in excess in this reaction. Mean and error bars in all graphs display the standard deviation from three technical repeats. **D.** Representative fluorescence curves showing a higher WT NTHL1 concentration leads to a higher initial reaction rate when APE1 (2nM) is present in the reaction with a Tg substrate (10nM).

Supplementary Figure 2

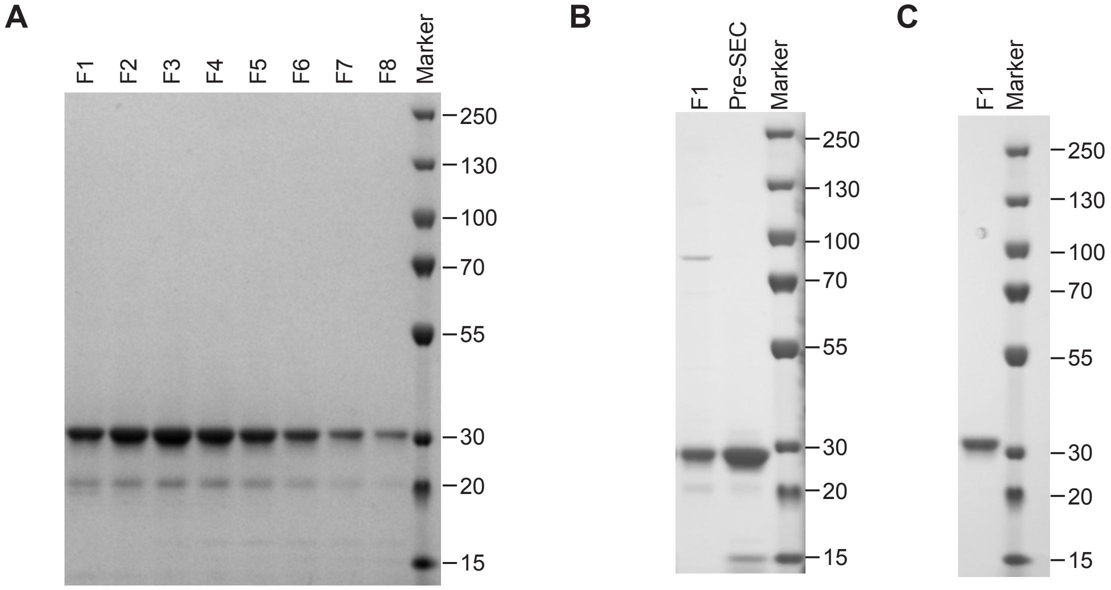

Supplementary Figure 2: Purity of recombinantly expressed NTHL1 and NTHL1 variant proteins A. Coomassie Blue stained SDS-PAGE of purified recombinant wild-type NTHL1 protein. Fractions (F) F1 to F8 post-purification were pooled and used for all biochemical experiments. Major band at ~30kDa corresponds to NTHL1. A minor band at 20kDa may correspond to SlyD, a common but non interfering host protein contaminant. **B.C.** Coomassie Blue stained SDS-PAGE of purified recombinant LK **(B)** and KQ **(C)** mutant NTHL1 proteins. F1 indicates the pooled protein used for biochemical experiments.

Supplementary Figure 3

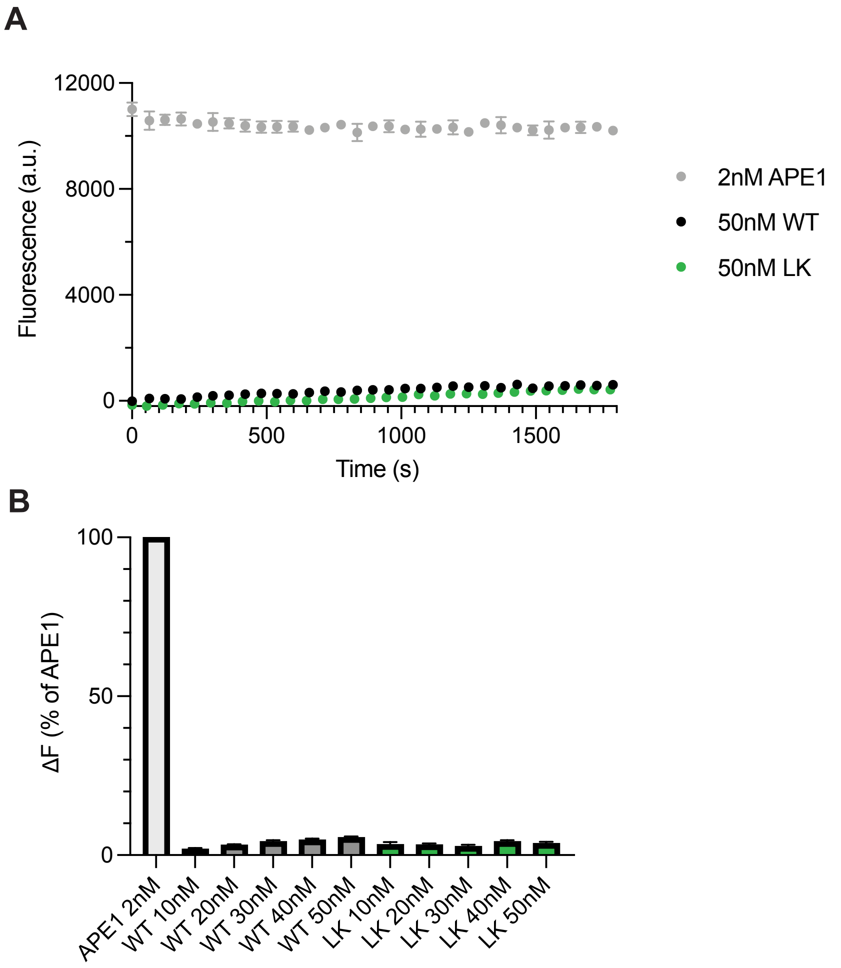

Supplementary Figure 3: APE1 but not WT or LK NTHL1 can catalyse efficient strand incision on a D-spacer AP site analog substrate. A. Representative fluorescent curves of APE1, 50nM WT or LK, over 1800 seconds (30 minutes) using 10nM of a D-spacer AP site analog substrate. Background fluorescence from 10nM of substrate alone was subtracted from all values. APE1 reaches maximum fluorescence within first measured time point. **B.** Bar plot showing the change in fluorescence (ΔF) over whole course of the monitored reaction (30 minutes), as depicted in (A). ΔF measured for 2nM APE1 alone was set as 100%. All other values are expressed as a percentage of ΔF APE1 alone. Error bars show means with standard deviations from three independent experiments.

Supplementary Figure 4

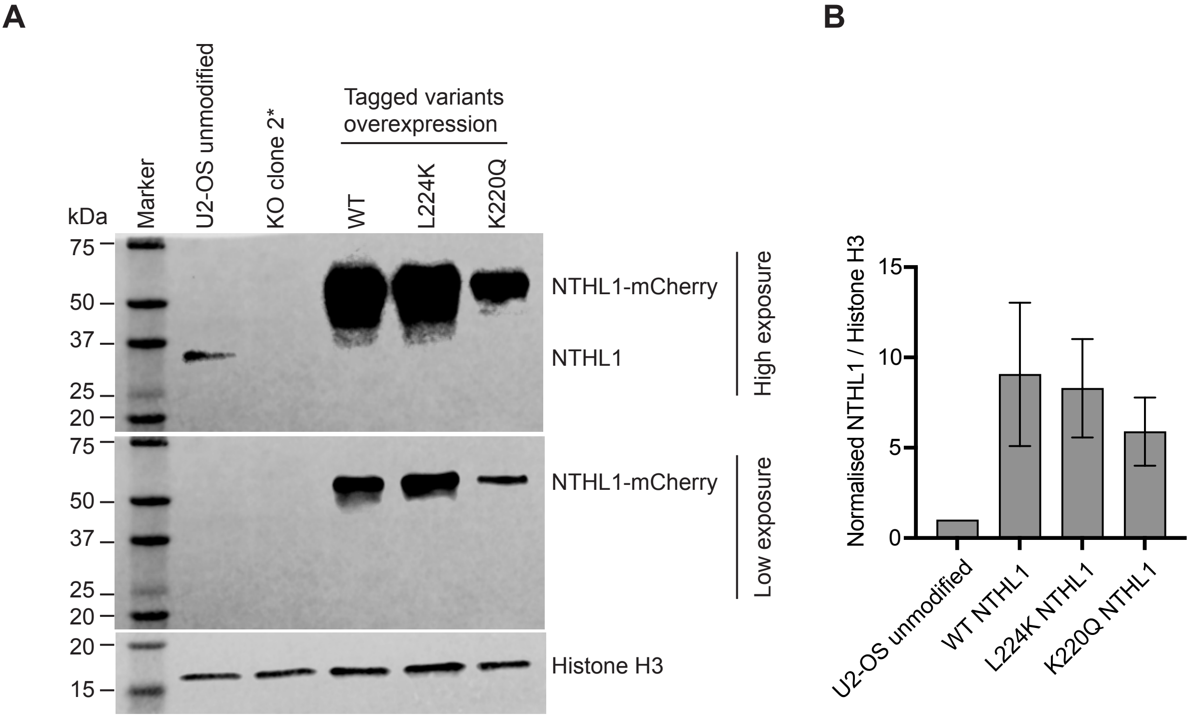

Supplementary Figure 4: Overexpression of tagged NTHL1 variants compared to the endogeneous level of untagged NTHL1 in unmodified U2-OS cells. A. Representative Western Blot. * is the KO clone used to re-overexpress the NTHL1-mCherry variant tagged contructs. **B.** Quantification of bands. The fold difference in NTHL1 expression was normalised to Histone H3 band intensity using ImageJ. Error bars indicate the standard deviation of the mean from the quantification from 3 independent experiments.

Supplementary Figure 5

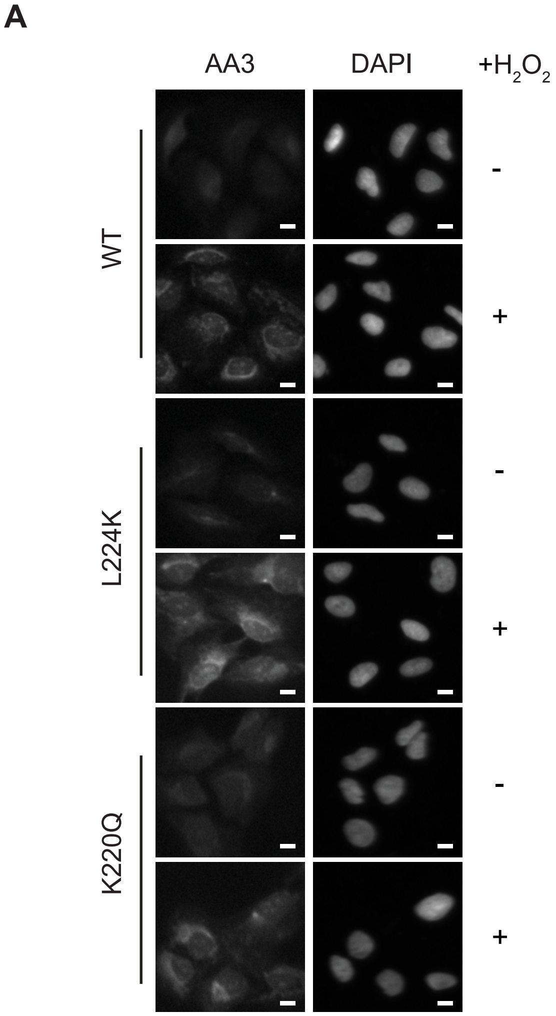

Supplementary Figure 5: Representative images from **Figure 3F**. Scale bar represents 5µm.

Supplementary Figure 6

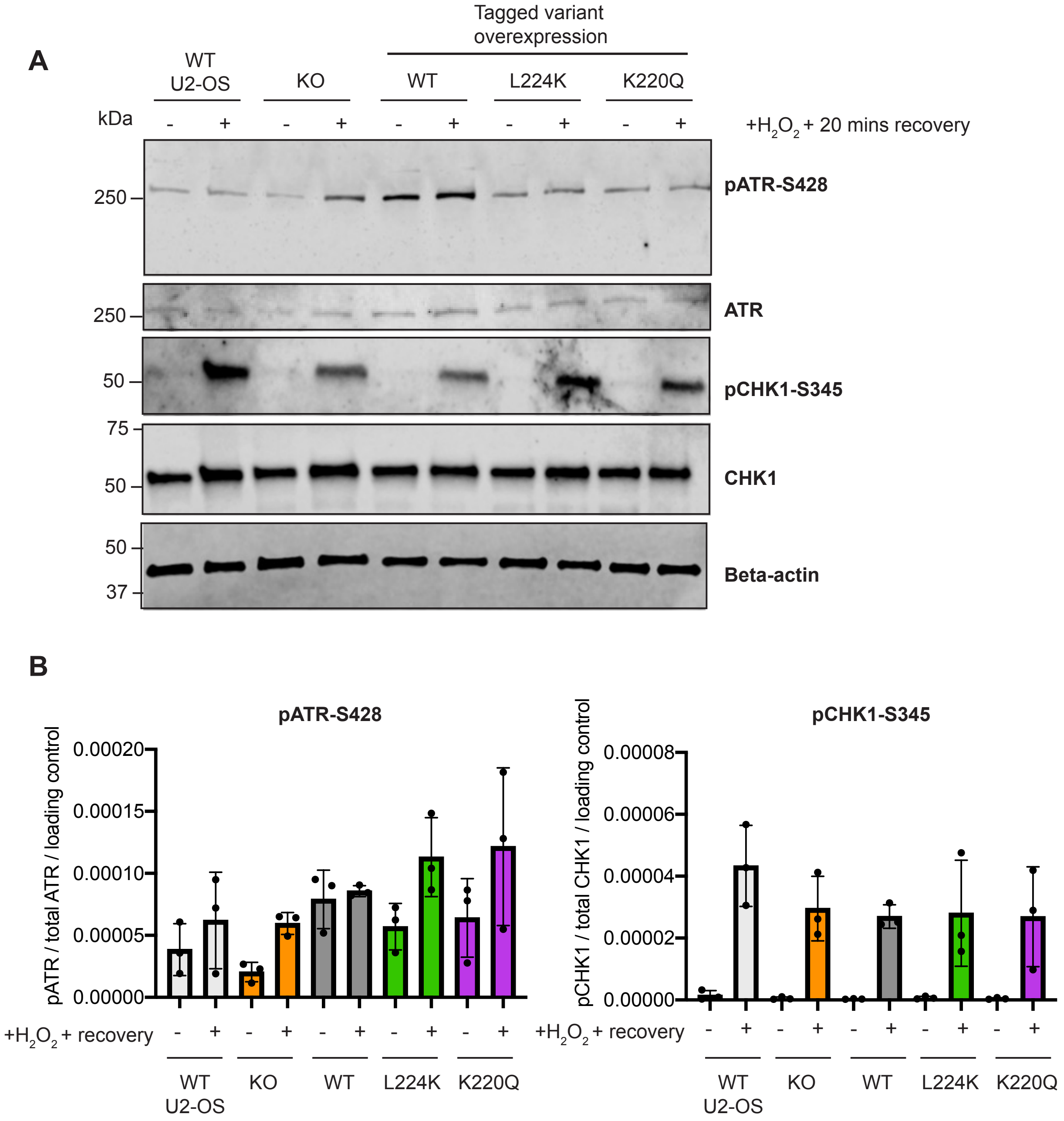

Supplementary Figure 6: H_2O_2 treatment induces pATR/pCHK1 signalling in U2-OS cells expressing endogeneous NTHL1, KO, or overexpressing variants of NTHL1. A. Indicated cell lines were treated with 500 µM H_2O_2 for 25 minutes, followed by a washout and 20 minute recovery period in fresh media before cell lysate preparation. Representative Western Blot with the indicated antibodies. **B.** Quantification of bands. The band intensity of phosphorylated proteins was normalised first to its total protein counterpart band intensity, then to loading control beta-actin. Mean and error bars indicate the standard deviation of the quantification from 3 independent experiments.

Supplementary Figure 7

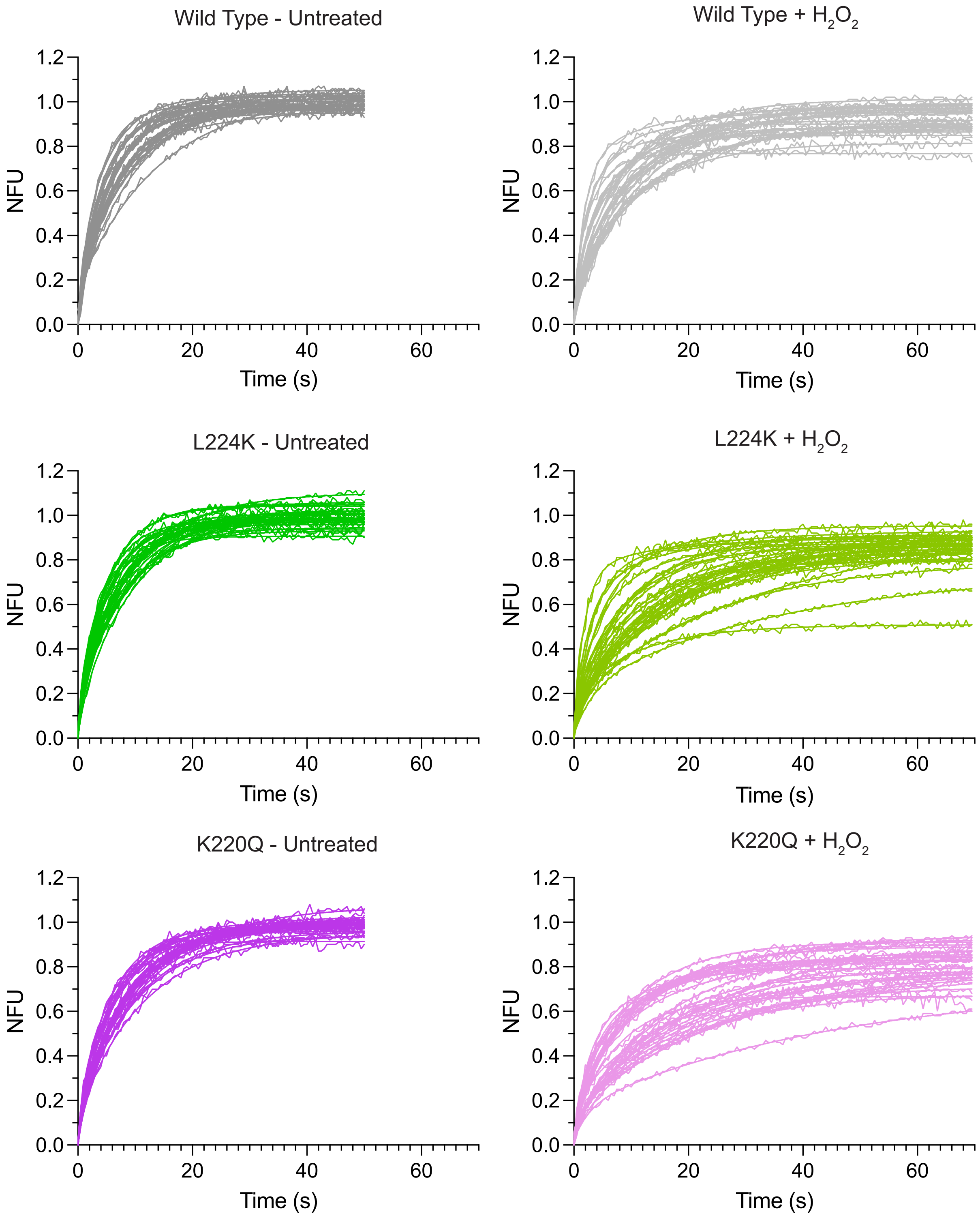

Supplementary Figure 7: Individual cell FRAP curves with two-phase association fit during oxidative stress. Treated cells display increased variability in recovery kinetics, reflecting the single-cell resolution of the assay. Individual FRAP curves used to calculate parameters shown in **Figure 5**. For conditions where all curves' plateau was reached by 50 seconds, then data after 50 seconds was omitted to avoid overfitting of the plateau and ensure accurate estimation of the slow phase half-life.

Supplementary Figure 8

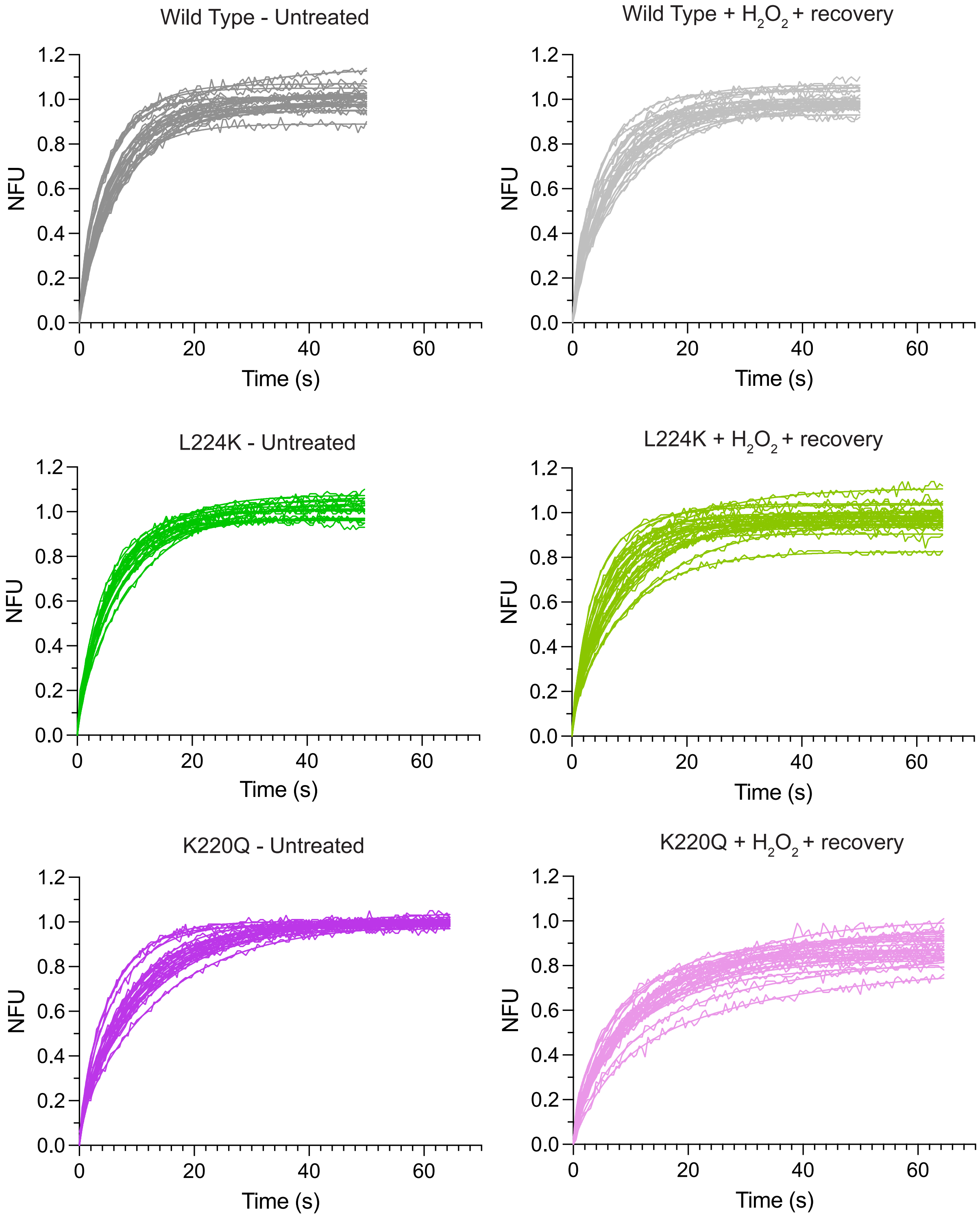

Supplementary Figure 8: Individual cell FRAP curves with two-phase association fit following a recovery period. Individual FRAP curves used to calculate parameters shown in **Figure 6**. For conditions where all curves' plateau was reached by 50 seconds, then data after 50 seconds was omitted to avoid overfitting of the plateau and ensure accurate estimation of the slow phase half-life.

Supplementary table 1 - Oligonucleotide sequences

Oligonucleotide	Sequence 5' to 3'	Source
Tg:A fluorescent strand	FCTGCCA**Tg**CACTGCGTCGACCTG	TriLink Biotech
Tg:A .2 quencher strand	CAGGTCGACGCAGTGATGGCAGDab	TriLink Biotech
U:A fluorescent strand	FCTGCCA**U**CACTGCGTCGACCTG	ADTBio
U.A quencher strand	CAGGTCGACGCAGTGATGGCAGDab	ADTBio
D-spacer:A fluorescent strand	FTCTG CCA **D**CA CTG CGT CGA CCT G	ADTBio
D-spacer:A quencher strand	CAG GTC GAC GCA GTG CTG GCA GTX	ADTBio

Where **Tg** = thymine glycol, F = FAM, Dab = dabcyl, **D** = D-spacer, X = BHQ, **U** = uracil

Supplementary table 2 – Guide RNA (gRNA) sequences

gRNA	Sequence 5' to 3'
NTHL1 sgRNA 1	GCCCCUCAAGGUGCCAGUCU
NTHL1 sgRNA 2	GCUUUCCGCGGACGCUUCAC
NTHL1 sgRNA 3	UGCAUCCUUUUUGUUCCUCA

Supplementary table 3 – Primers used in study

Primer	Sequence 5' to 3'
NTHL1_sg123_fw	CCAAAAGCCACCGGGTAGAA
NTHL1_sg123_re	CCACATACCCCAGACTGCAG
NTHL1_sg123_seq	AAAAGCCACCGGGTAGAAAGAAAACAAG

Supplementary Table 4 – Antibodies used in study

**Antibody**	**Dilution and application** (WB = Western Blot / IF = immunofluorescence)	**Source and catalog number**
XRCC1, monoclonal mouse	1:500, IF	Abcam, ab1838
Phospho-Histone H2A.X (Ser139), polyclonal rabbit	1:1000, IF/WB	Cell Signalling Technology, #2577
NTHL1, monoclonal mouse	1:1000, WB	Abcam, ab70726
ATR, polyclonal rabbit	1:1000, WB	Cell Signalling Technology, #2790
Phospho-ATR (Ser428), rabbit	1:1000, WB	Cell Signalling Technology, #2853
Chk1, monoclonal mouse	1:1000, WB	Cell Signalling Technology, #2360
Phospho-Chk1 (Ser345), rabbit	1:1000, WB	Cell Signalling Technology, #2341
Histone H3, rabbit	1:5000, WB	Abcam, ab1791
Beta-actin, monoclonal mouse	1:5000, WB	Abcam, ab6276
IRDye 800CW donkey anti-mouse IgG	1:10,000, WB	Licor, #925-32212
IRDye 800CW donkey anti-rabbit IgG	1:10,000, WB	Licor, #926-32213
Peroxidase-conjugated AffiniPure Donkey Anti-Rabbit IgG (G+L)	1:10,000, WB	Jackson ImmunoResearch, #711-035-152
